# Dynamic evolution of the GnRH receptor gene family in vertebrates

**DOI:** 10.1186/s12862-014-0215-y

**Published:** 2014-10-25

**Authors:** Barry L Williams, Yasuhisa Akazome, Yoshitaka Oka, Heather L Eisthen

**Affiliations:** Department of Zoology and BEACON Center for the Study of Evolution in Action, Michigan State University, East Lansing, Michigan USA; Department of Microbiology and Molecular Genetics, Michigan State University, East Lansing, Michigan USA; Department of Biological Sciences, School of Science, University of Tokyo, Bunkyo-ku, Tokyo Japan

**Keywords:** Amphibians, Hormones, Mammals, Peptides, Reproduction

## Abstract

**Background:**

Elucidating the mechanisms underlying coevolution of ligands and receptors is an important challenge in molecular evolutionary biology. Peptide hormones and their receptors are excellent models for such efforts, given the relative ease of examining evolutionary changes in genes encoding for both molecules. Most vertebrates possess multiple genes for both the decapeptide gonadotropin releasing hormone (GnRH) and for the GnRH receptor. The evolutionary history of the receptor family, including ancestral copy number and timing of duplications and deletions, has been the subject of controversy.

**Results:**

We report here for the first time sequences of three distinct GnRH receptor genes in salamanders (axolotls, *Ambystoma mexicanum*), which are orthologous to three GnRH receptors from ranid frogs. To understand the origin of these genes within the larger evolutionary context of the gene family, we performed phylogenetic analyses and probabilistic protein homology searches of GnRH receptor genes in vertebrates and their near relatives. Our analyses revealed four points that alter previous views about the evolution of the GnRH receptor gene family. First, the “mammalian” pituitary type GnRH receptor, which is the sole GnRH receptor in humans and previously presumed to be highly derived because it lacks the cytoplasmic C-terminal domain typical of most G-protein coupled receptors, is actually an ancient gene that originated in the common ancestor of jawed vertebrates (Gnathostomata). Second, unlike previous studies, we classify vertebrate GnRH receptors into five subfamilies. Third, the order of subfamily origins is the inverse of previous proposed models. Fourth, the number of GnRH receptor genes has been dynamic in vertebrates and their ancestors, with multiple duplications and losses.

**Conclusion:**

Our results provide a novel evolutionary framework for generating hypotheses concerning the functional importance of structural characteristics of vertebrate GnRH receptors. We show that five subfamilies of vertebrate GnRH receptors evolved early in the vertebrate phylogeny, followed by several independent instances of gene loss. Chief among cases of gene loss are humans, best described as degenerate with respect to GnRH receptors because we retain only a single, ancient gene.

**Electronic supplementary material:**

The online version of this article (doi:10.1186/s12862-014-0215-y) contains supplementary material, which is available to authorized users.

## Background

Gonadotropin releasing hormone (GnRH) is a decapeptide produced by neurons in the hypothalamic-preoptic area in vertebrates; it causes pituitary gonadotrope cells to release follicle stimulating hormone and luteinizing hormone. Although redundant mechanisms ensure that many physiologically important functions will be carried out, the hypothalamic-pituitary GnRH signaling system is irreplaceable: animals in which the GnRH-producing neurons do not develop properly, or in which the GnRH receptor is mutated or knocked out, do not reach sexual maturity [1-4]. Thus, although GnRH is involved in additional physiological functions, maintenance of the reproductive GnRH signaling system is subject to strong stabilizing selection pressure.

GnRH receptors are part of the superfamily of G-protein coupled receptors (GPCRs), which generally consist of seven membrane-spanning segments, an extracellular N-terminal domain, and a cytoplasmic C-terminal domain. The first GnRH receptor genes sequenced exhibited expression restricted to the pituitary gland in mice and rats [5-7], and were notably unusual GPCRs in that they lack the cytoplasmic C-terminal domain, terminating near the inner surface of the cell membrane. This feature is unexpected, as the cytoplasmic tail of GPCRs plays a key role in desensitization and internalization of the receptor [8].

The second mammalian GnRH receptor discovered is expressed more widely within the nervous system and possesses the typical cytoplasmic C-terminal domain [9]. Additional GnRH receptor genes have since been identified in other vertebrates; for example, frogs possess three [10] and some teleost fishes possess as many as five [11]. None of these receptors is closely related to the original mammalian GnRH receptor, which lacks a cytoplasmic tail. Thus, the evolutionary origin and diversification of GnRH receptors has been a subject of controversy [12-15].

As an extension of our research examining the modulatory effects of GnRH on olfactory system function in salamanders [16,17], we sought to clone the GnRH receptors from axolotls (*Ambystoma mexicanum*), a model salamander species. Here we show the identification of three GnRH receptor genes from axolotls, the first from urodele amphibians. The presence of three GnRH receptors was consistent with ortholog composition in other amphibians. To understand the types of GnRH receptors present in axolotls from an evolutionary perspective, we carried out phylogenetic analyses of GnRH receptor genes, as well as probabilistic peptide homology searches. We found that vertebrate GnRH receptor genes were composed of five subfamilies, different from previous classifications [13-15], and clarified the evolutionary relationships among and within the subfamilies. Finally, we found that mammalian-type GnRH receptors, which lack the C-terminal cytoplasmic tail, were present in the genomes of a skate (*Leucoraja erinacea*), a cartilaginous fish, the chimaera (*Callorhinchus milii*), and a lobe-finned fish, the coelacanth (*Latimeria chalumnae*). This result contradicts long-standing assumptions that the loss of the cytoplasmic tail was a rapid evolutionary adaptation key to the unique reproductive biology of mammals [8, but see 15,18-29]. Based on these results, we present a new evolutionary hypothesis for the origin and diversification of vertebrate GnRH receptors.

## Results

### GnRH receptors in axolotls

Using primers designed based on sequence conservation of three GnRH receptors cloned in frogs, as well as selected sequences from teleosts, we isolated and sequenced three GnRH receptor cDNAs from axolotls. These are the first GnRH receptors identified from salamanders.

**Figure 1:**
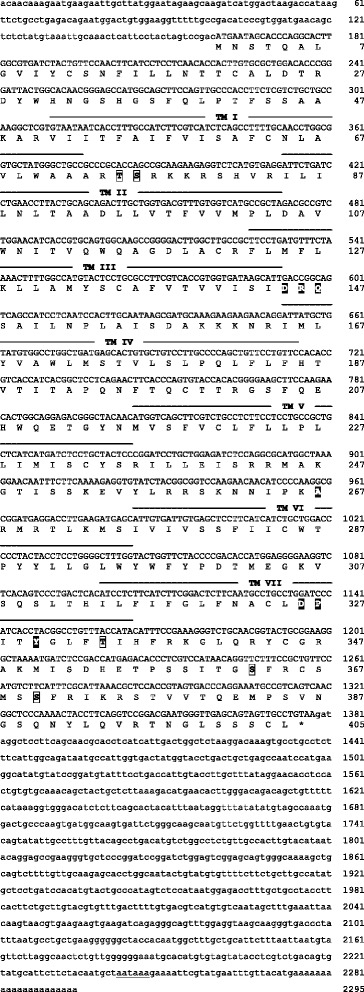
**cDNA and deduced amino acid sequence of the GnRH receptor IIa-2 gene in axolotls.** Within the cDNA sequences, lowercase letters indicate untranslated regions and uppercase letters indicate the open reading frame; underlining indicates the polyadenylation site. Transmembrane domains were predicted using the HMMTOP 2.0 server [30] and are indicated above the relevant portion of the sequence. Putative G-protein interaction sites are inferred based on homology with *Xenopus laevis* [23] and are indicated with a black background. Transparent boxes indicate putative phosphorylation sites (>80% probability) predicted using the NetPhosK server [31].

**Figure 2:**
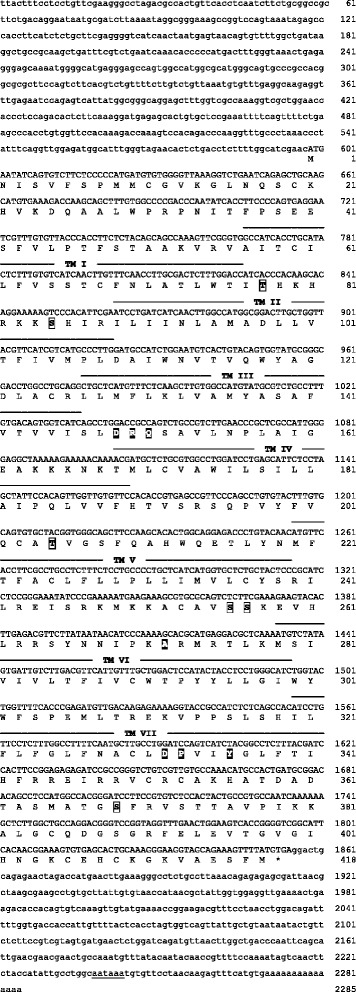
**cDNA and deduced amino acid sequence of the GnRH receptor IIa-3 gene in axolotls.** Analysis and formatting as described in Figure 1.

**Figure 3:**
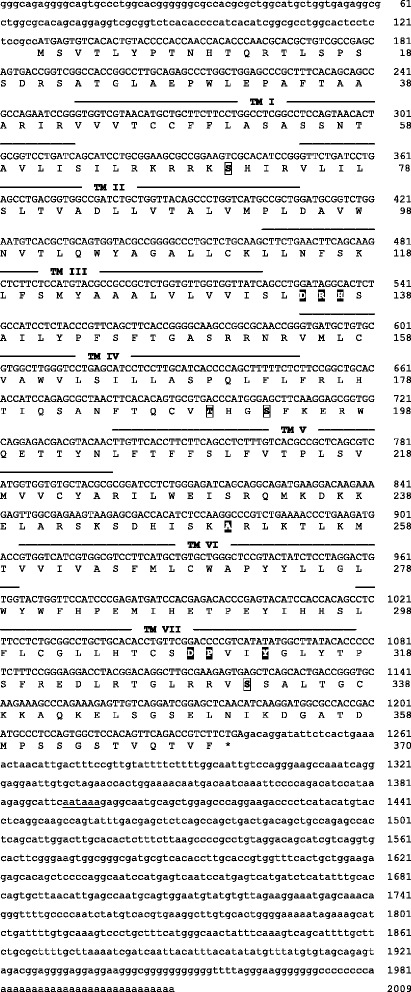
**cDNA and deduced amino acid sequence of the GnRH receptor IIb gene in axolotls.** Analysis and formatting as described in Figure 1.

**Figure 4:**
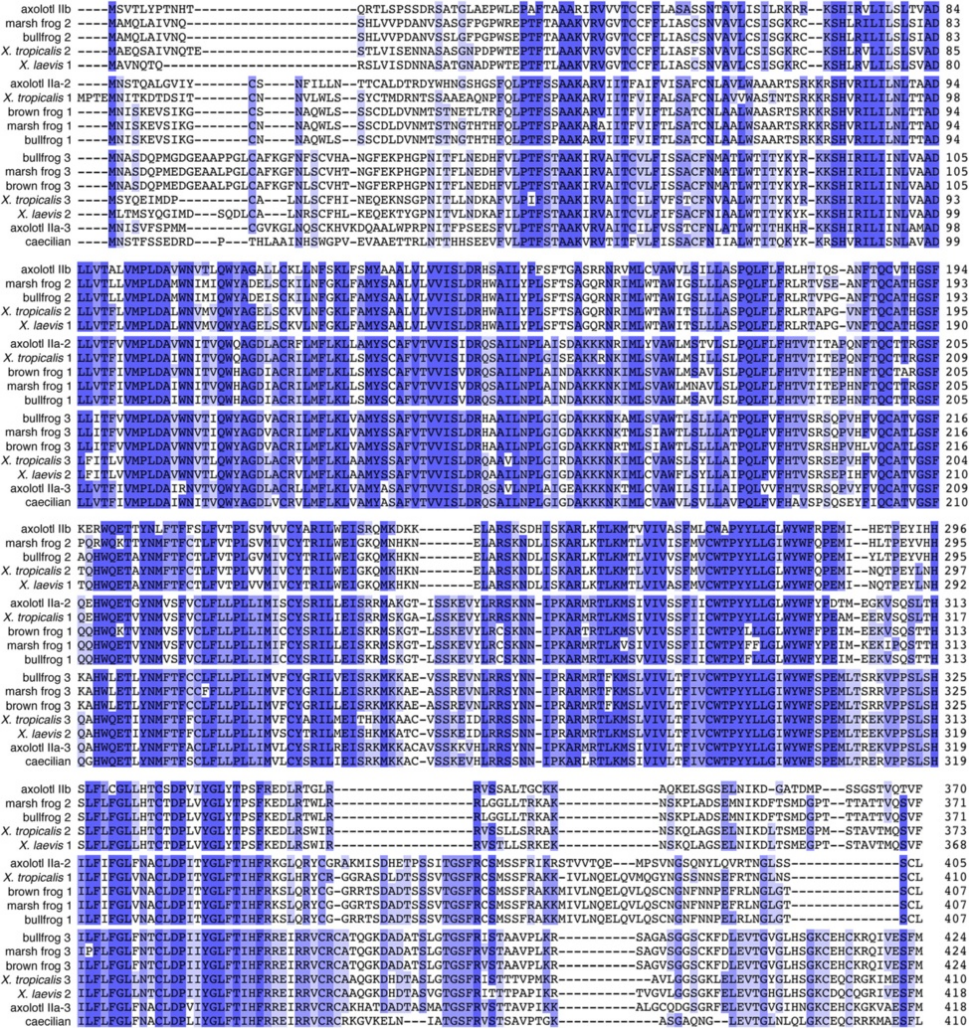
**Comparison of deduced amino acid sequences for the three amphibian GnRH receptor genes, aligned using MUSCLE** **[** 32 **]** **.** Each of the three genes identified in frogs has a clear ortholog in axolotls. Amino acids that are highly conserved (>80% sequence identity) are indicated with the darkest shading; lighter shading indicates 60-80% identity; the lightest shading indicates 40-60% identity; and a white background indicates low conservation (<40% identity). Latin names of species and GenBank accession numbers for sequences are provided in Additional file 1: Table S1.

**Figure 5:**
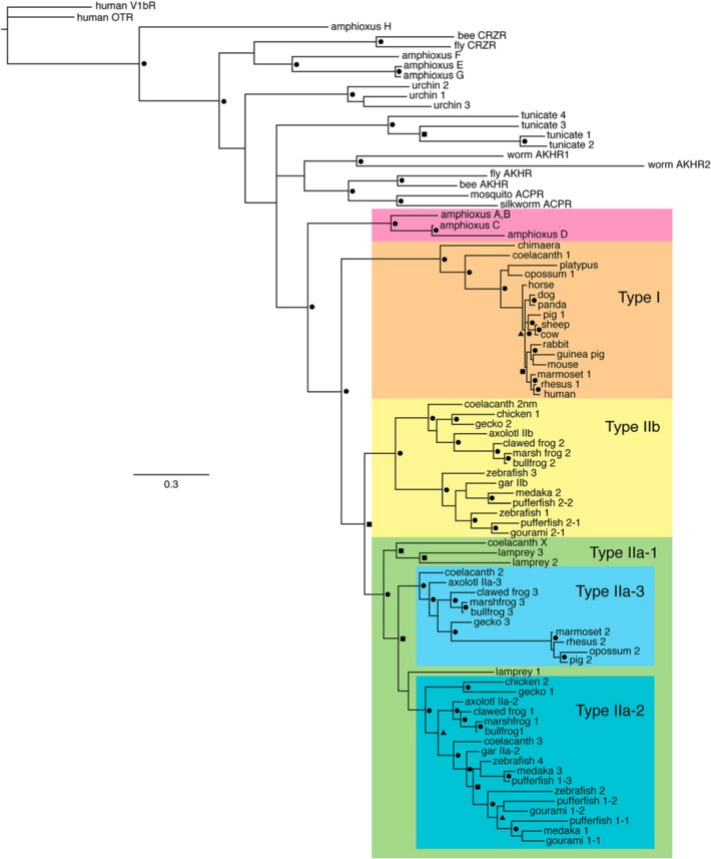
**Phylogram from Bayesian analyses depicting the evolutionary relationships among the genes encoding receptors for GnRH and other peptides.** Two amphioxus sequences, **A** and **B**, differ by a single amino acid; thus, only one was included in the analysis. Colored backgrounds emphasize strongly-supported, monophyletic subfamilies of GnRH receptors*.* Symbols indicate three categories of support value: triangles, posterior probability of 0.90-0.95; squares, posterior probability of 0.95-0.99; circles, posterior probability of 1.0. ACPR = adipokinetic hormone/corazonin-related peptide receptor; AKHR = adipokinetic hormone receptor; CRZR = corazonin receptor; OTR = oxytocin receptor; V1bR = type 1b vasopressin receptor. The scale bar depicts a branch length corresponding to 0.3 amino acid substitutions per site. Latin names of species and accession numbers for sequences are provided in Additional file 1: Table S1.

Nucleotide and predicted amino acid sequences of the three receptors are illustrated in Figures 1, 2, and 3. Predicted locations of the seven membrane-spanning regions, as well as predicted sites of G-protein interaction and phosphorylation, are also shown. As illustrated in Figure 4, the sequences obtained from axolotls exhibit high sequence similarity with those from other amphibians. Three ‘subtypes’ of receptors are apparent (see also Figure 5), and we did not detect additional paralogs or splice variants. Based on the results described below, we determined that two of the receptors fall into the category that Roch et al. [14] named “Type IIa” and that the third receptor falls into their “Type IIb” category. We have therefore named the three axolotl GnRH receptors IIa-2, IIa-3, and IIb, with GenBank accession numbers KF499141, KF499142, and KF499143, respectively.

### Evolutionary relationships of the GnRH receptors

The evolutionary relationships among GnRH receptors and their near relatives are illustrated in Figure 5. Our Bayesian analysis revealed that vertebrate GnRH receptors form a monophyletic group that excludes all receptors sequenced from other organisms, including some that have previously been identified as GnRH receptors. Within the vertebrate GnRH receptor clade, the most ancestral node separates the Type I and Type II receptors. Naming conventions for this gene family have not been consistent, and we have named major clades within the family following the conventions outlined in the phylogenetic analyses by Roch and colleagues [14]. To facilitate cross-indexing among naming conventions, we have also listed each gene using its original name.

**Figure 6:**
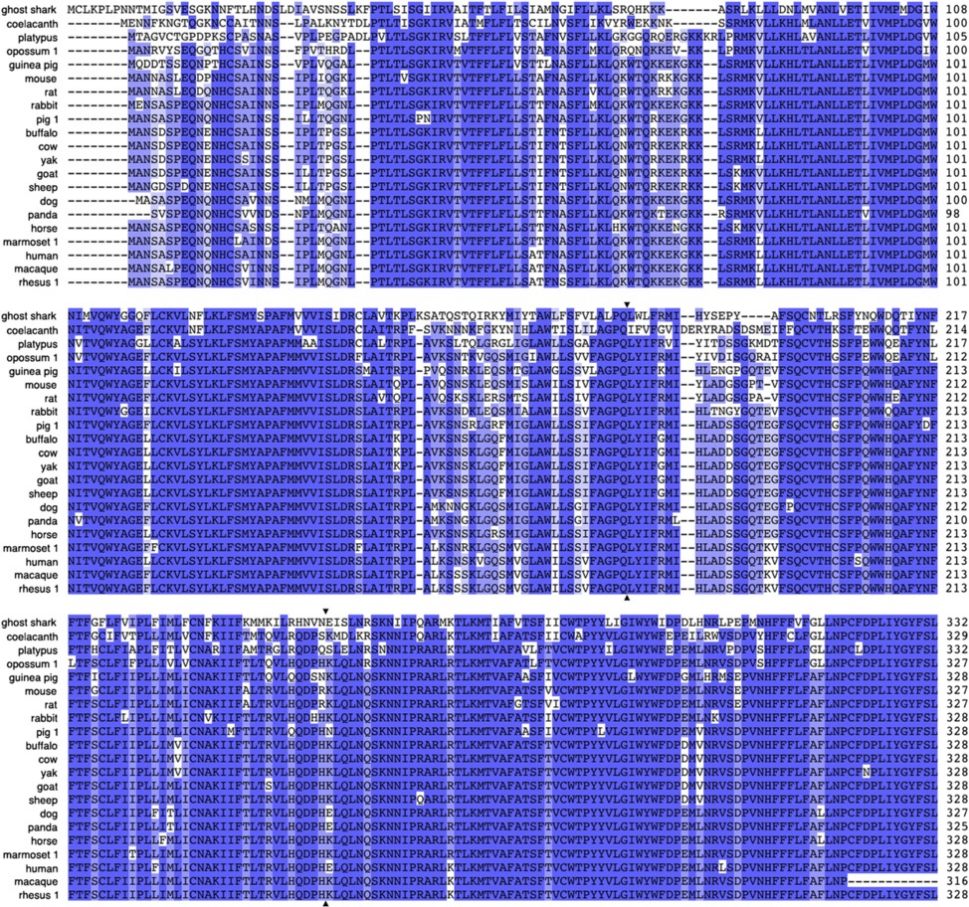
**Complete open reading frame sequences for Type I GnRH receptors, aligned using MUSCLE** **[** 32 **]** **.** The Type I receptors identified in chimaeras and coelacanths (uppermost sequences) share high sequence similarity with those from mammals as well as identical intron-exon boundaries (arrowheads) and the lack of a cytoplasmic C-terminal domain. Amino acids that are highly conserved (>80% sequence identity) are indicated with the darkest shading; lighter shading indicates 60-80% identity; the lightest shading indicates 40-60% identity; and a white background indicates low conservation (<40% identity). Transmembrane domains were predicted using HMMTOP 2.0 [30] and are indicated with dark lines surrounding the relevant portion of the sequences. Latin names of species and accession numbers for sequences are provided in Additional file 1: Table S1.

Although the Type I receptors had previously been known only from mammals, we found full length sequences of this receptor type in the genomes of a coelacanth (*Latimeria chalumnae*) and a chimaera (the elephant shark *Callorhinchus milii*), as well as partial sequence orthologs in the genome of the little skate (*Leucoraja erinacea*)(see Additional file 1: Table S1 and below). Like the Type I receptor in mammals, those in coelacanths and chimaera lack the cytoplasmic tail, terminating at the same amino acid. The putative Type I receptors also share the same intron-exon boundaries, strongly indicative of orthology. Figure 6 illustrates the alignment of these sequences and the locations of exon boundaries.

The most ancestral node within the Type II receptor clade separates two large clades, which Roch et al. [14] designated Type IIa and IIb. Our analyses indicate that the Type IIa receptors can be further subdivided into three distinct clades, which we call Type IIa-1, IIa-2 and IIa-3 (Figure 5). Full-length Type IIa-1 receptor genes used in phylogenetic analyses were limited to those from lampreys and coelacanths. This subfamily was not monophyletic, but its delineation was based on results from probabilistic homology searches using peptide sequences, detailed below. The Type IIa-2 receptors are present in teleosts, coelacanths, amphibians, and reptiles, including birds, but appear to have been lost in the common ancestor to mammals. In addition, two copies of the Type IIa-2 GnRH receptor are present in teleosts, indicating the retention of a duplicate copy in a teleost ancestor. A third Type IIa-2 receptor is unique to pufferfish, which indicates at least one additional lineage specific duplication event (Figure 5). The Type IIa-3 receptor is present only in coelacanths, amphibians, reptiles, and some mammals. The relatively recent common ancestry between the Type IIa-2 and IIa-3 receptors combined with the nested taxonomic distribution for Type IIa-3 receptors suggests it is the youngest subfamily. Topological position and taxonomic distribution indicate the Type IIa-3 subfamily arose through duplication of a Type IIa-2 receptor in early sarcopterygians. The absence of Type IIa-3 receptors in birds and multiple clades of mammals indicates additional lineage specific losses of this receptor subtype.

Finally, the Type IIb GnRH receptor subfamily is present in all vertebrate clades surveyed, with the exception of mammals. As observed with the Type IIa-2 receptors, a pair of Type IIb GnRH receptor genes was present in both zebrafish and pufferfish. Ancestral teleosts appear to have harbored only the Type IIa-2 and IIb subfamilies. Both subfamilies are typically single copy, but each was found as a pair of duplicate genes in teleosts, consistent with the teleost genome duplication event. The single copy genes for the Type IIb subfamily in medaka and gourami could be the result of independent gene loss. However, many teleost Type IIb receptors were identified on small contigs that lacked syntenic neighboring genes, so gene absence could be a technical artifact of genes missing from each genome database.

Additional results from BLAST searches of the chimaera, spotted gar (*Lepisosteus oculatus*), little skate, and lamprey (*Petromyzon marinus*) genomes also resulted in the identification of partial sequences (less than 60 amino acids) for putative homologs. The small size of these sequences precluded their use in phylogenetic analyses. These four species are found at important topological positions with respect to the origin of GnRH receptor clades, but the presence or absence of homologs based only on sequences used for phylogenetic analyses constituted a potentially biased sample. Apparent gene absence could be due sub-sampling during targeted cloning using degenerate PCR primers. Apparent absence could also be a technical artifact from low sequence coverage, consistent with the observation that the genome assemblies for these taxa largely comprise small contigs. To address this issue, we used probabilistic methods to determine sequence homology for short peptide sequences. Specifically, we constructed Hidden Markov Models (HMM) corresponding to four different sequence regions nested within a canonical GnRH receptor, where the size and location of each region corresponded with peptides consistently identified in BLAST searches (termed TM1-4, TM4-5, TM6 and TM6-7 in Table 1, Additional file 2: Table S2 and Additional file 3: Table S3). The HMM profiles were further refined into ‘types’ based on the taxonomic subset of input GnRH receptor sequences used for HMM construction with HMMER v. 3.1 [33].

**Table 1:** **Skate and lamprey gene homology determined with GnRH receptor type-specific HMM profiles of protein motifs using HMMER** [34]

	**Little skate genome**					
Sequence ID	AESE012567234.1	AESE011658775.1	AESE011105720.1	AESE010056425.1	AESE011520245.1	AESE012567234.1
Domain Homology‡	TM1 to TM4	TM1 to TM4	TM1 to TM4	TM6 to TM7	TM6 to TM7	TM6 to TM7
HMM-Type I	4.7E-28 (107.9)	1.1E-34 (129.2)	**3.4E-48 (173.2)**	1.3E-19 (79,9)	9.3E-29 (109.2)	**1.8E-38 (140.4)**
HMM-Type IIa-1	**7.1E-50 (178.5)**	2.4E-60 (212.4)	3.9E-43 (127.5)	**5.5E-33 (122.6)**	3.5E-33 (123.2)	6.73E-3 (119.1)
HMM-Type IIa-2	1.7E-47 (107.7)	**8.5E-68 (236.5)**	1.5E-31 (199.1)	1.0E-25 (99.5)	**1.3E-40 (147.3)**	1.4E-30 (115.1)
HMM-Type IIa-3	5.0E-41 (149.8)	7.2E-64 (223.8)	2.2E-32 (121.8)	3.6E-25 (97.7)	5.1E-39 (142.1)	9.3E-28 (106.0)
HMM-Type IIb	8.1E-41 (149.0)	2.1E-50 (180.0)	2.9E-35 (131.0)	1.6E-23 (92.2)	2.5E-21 (117.2)	2.1E-32 (120.6)
	**Lamprey genome**					
Contig ID	22569.4_6 (586)	42790.2_1 (476)	31731.1_3 (265)	30359.1_1 (909)	36401.1_5 (1441)	
Domain Homology‡	Tm1 to Tm4	Tm1 to Tm4	Tm1 to Tm4	Tm6 to Tm7	Tm6 to Tm7	
HMM-Type I	1.2E-44 (138)	1.0E-33 (103)	1.9E-23 (69)	3.7E-36 (110)	7.2E-30 (90)	
HMM-Type IIa-1	**3.4E-75 (237)**	**2.5E-59 (186)**	**1.7E-41 (128)**	**6.7E-48 (138)**	**3.6E-45 (138)**	
HMM-Type IIa-2	7.3E-61 (191)	1.2E-49 (154)	7.3E-34 (103)	4.5E-39 (119)	1.2E-32 (98)	
HMM-Type IIa-3	2.9E-59 (185)	6.9E-47 (145)	3.0E-32 (97)	4.9E-38 (116)	1.3E-32 (98)	
HMM-Type IIb	2.5E-54 (169)	9.7E-46 (141)	2.5E-31 (95)	9.5E-37 (111)	3.0E-33 (100)	

Numbers represent e-values (with bit scores shown in parentheses) resulting from each HMMER search, in which the HMM profile was constructed with sequence input limited to GnRH receptors from each respective subclade; boldface font indicates the best e-value within each column. The HMM profiles were then used as queries against either the little skate (*Leucoraja erinacea*) or lamprey (*Petromyzon marinus*) genome. Reciprocal searches in which each sequence was used as a query against all HMM profiles (those constructed in this work in addition to all HMM models in the Pfam database) were congruent (Additional file 2: Table S2).

‡ Domain homology indicates the approximate physical position of the HMM with respect to the transmembrane (TM) domains in Figure 10.

Type I receptor homologs were not identified in the chimaera genome assembly. However, two putative exons of the Type I receptor were identified in the little skate genome assembly (Table 1 and Additional file 2: S2). In addition, results from HMM searches indicate a Type IIa-1 ortholog in the chimaera (Additional file 3: Table S3) in addition to the Type I, IIa-1 and IIa-2 orthologs in the little skate (Table 1). Only Type IIb and IIa-2 GnRH receptor orthologs were identified in the gar genome assembly, consistent with results from BLAST searches. Three GnRH receptor homologs were identified in the lamprey genome, which is consistent with previous cloning efforts [35]. The phylogenetic analyses produced an ambiguous classification for the lamprey GnRH receptor genes because support was low (posterior probability = 0.67) for the placement of the lamprey type 1 gene within the Type IIa clade (Figure 5). Conversely, all three lamprey genes matched the Type IIa-1 subclade HMMs significantly better than alternative subclades (Table 1).

All homologs identified using HMMER v. 3.1 resulted from matches with two HMMs, where each matched a sequence found on a unique contigs. Some of the contig were large enough to harbor an additional 30,000 base pairs beyond the sequence match. Because the two HMM profiles were constructed from nearly adjacent segments of the mature protein, two sequence matches across contigs indicates that each HMM profile likely corresponded with an exon. Finally, the orthology designations were consistent between reciprocal search approaches: either the HMM profiles were used as queries to search genome assemblies, or sequences were used as queries to search an HMM database of profiles from this work in addition to the 18,523,877 HMM profiles from the Pfam database (release 27.0 [33]; Additional file 2: Table S2 and Additional file 3: Table S3).

As illustrated in Figure 5, four receptor sequences from the amphioxus (*Branchiostoma floridae*) genome may represent GnRH receptors, but all four genes formed a monophyletic clade ancestral to vertebrate GnRH receptors. Similarly, four sequences obtained from the genome of a tunicate (*Ciona intestinalis*) and three genes from the sea urchin (*Strongylocentratus purpuratus*) genome, which were previously characterized as GnRH receptors [36], each form strongly supported monophyletic clades. Thus, the amphioxus*,* tunicate, and urchin GnRH receptor paralogs each appear to be the result of lineage-specific duplication events of a single ancestral gene. Homologs from a variety of invertebrates that had previously been identified as receptors for adipokinetic hormone (AKH) and adipokinetic hormone/corazonin-related peptide (ACP) formed monophyletic clades, the placement of which was uncertain. However, despite the lack of support for some ancestral branches in the GnRH receptor tree, inspection of the tree bipartitions sampled during the Markov chain Monte Carlo (MCMC) process in Bayesian analyses indicated that the tunicate clade was always ancestral to the amphioxus ‘A through D’ clade, and the sea urchin clade was always ancestral to tunicates. Thus, the low support for the placement of some clades at the base of the GnRH receptor phylogeny results from inconsistency with respect to placement of the AKH and ACP receptor clades.

**Figure 7:**
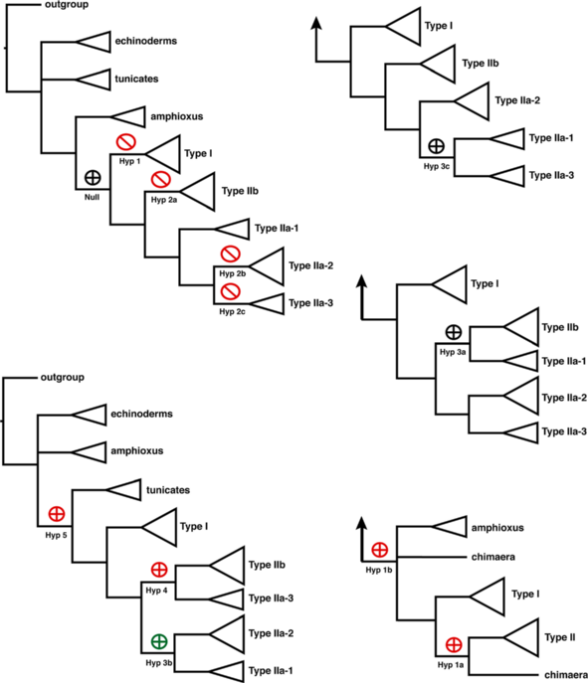
**Results of constrained topology tests using a stepping-stone model for Bayes Factor analyses** **[** 37 **]** **.** The shape of each symbol indicates the type of topology test used: plus inside a circle indicates a positive constraint, and slash inside a circle indicates a negative constraint. The color of each symbol indicates whether the constrained topology was significantly worse (red), better (green), or not significantly different from (black) the null hypothesis. More than one test is symbolized on each tree and constraints were applied only to the branch indicated; ancestral and derived branches were not constrained. Monophyletic clades from Figure 5 are summarized as triangles, but an individual sequence for the chimaera Type I receptor is listed separately in some cases. Positive constraints (circled plus) forced monophyly for the labeled branch and negative constraints (circled diagonal line) prohibited monophyly for the labeled branch. Arrows at the base of trees are symbols that summarize all ancestral clades.

We used constrained topology tests [37] to test various hypotheses concerning the relationships depicted in Figure 5. Table 2 and Figure 7 depict the hypotheses as well as the results from constrained topology tests using Bayes Factor. Results were generally consistent across three topological testing methods that include Bayes Factor, the “*Approximately Unbiased*” test, and the Shimodaira-Hasegawa test (Table 2 and Figure 7) [37-39]. The monophyly of subfamilies Type I, IIb, IIa-1, and IIa-2 was supported by Bayes Factor tests because negative constraints to prohibit the monophyly of each subfamily resulted in trees with significantly lower likelihood scores (hypotheses 1, 2a, 2b, and 2c, respectively; Table 2 and Figure 7). As an alternative method to test whether the chimaera receptor is a member of the Type I subfamily, positive constraint tests that forced the chimaera Type I GnRH receptor sequence to be either derived (hypothesis 1a) or ancestral (hypothesis 1b) to the Type I subfamily were tested using the Approximately Unbiased (AU) and Shimodaira-Hasegawa (SH) tests and rejected. The position of the Type IIa-1 subfamily as monophyletic with either the Type IIb, IIa-2, and IIa-3 subfamilies was tested using positive topological constrains (hypotheses 3a, 3b, and 3c, respectively). Monophyly of the Type IIa-1 and IIb subfamilies was not rejected using the Bayes Factor, but was rejected as very highly significant for both the AU and SH tests. Hypotheses testing the monophyly of the Type IIa-1 subfamily with either the Type IIa-2 or IIa-3 subfamilies (hypotheses 3b and 3c, respectively) were not rejected, except for two instances. The AU test rejected the Type IIa-1 and IIa-3 subfamilies as sister clades and the Bayes Factor test forcing monophyly of Type IIa-1 plus IIa-2 subfamilies (hypothesis 3b) was significantly more likely than the null hypothesis. The significance of the latter result is likely biased due to the different number of topologies explored between the null and test constraint hypotheses [37]. Unlike the uncertainty for placement of the Type IIa-1 subfamily, positive constraint tests that forced monophyly of the Type IIb plus IIa-3 subfamilies (hypothesis 4) were consistently rejected. Finally, positive constraint topology tests that forced the tunicate GnRH receptors to be derived relative to the amphioxus homologs (hypothesis 5) were strongly rejected by the Bayes Factor test but were non-significant for the AU and SH tests.

**Table 2:** **Results of constrained topology tests using Bayesian and maximum likelihood methods**

**Model**	**Constraint**	**Topology test**		
		Bayes factor	AU test	SH test
Hyp 1: Type I GnRH receptors are not monophyletic	Negative	15.300**	N/A	N/A
Hyp 1a: Chimaera Type I receptor is within the monophyletic Type II subfamily	Positive	N/A	> 0.001	0.013
Hyp 1b: Chimaera Type I receptor is ancestral to vertebrate receptors	Positive	N/A	0.002	0.013
Hyp 2a: Type IIb receptors are not monophyletic	Negative	12.970**	N/A	N/A
Hyp 2b: Type IIa-2 receptors are not monophyletic	Negative	13.280**	N/A	N/A
Hyp 2c: Type IIa-3 receptors are not monophyletic	Negative	15.300**	N/A	N/A
Hyp 3a: Type IIa-1 and IIb subfamilies are monophyletic to the exclusion of all other clades	Positive	2.080**	> 0.001	> 0.001
Hyp 3b: Type IIa-1 and IIa-2 subfamilies are monophyletic to the exclusion of all other clades	Positive	(−5.660)**	0.387	0.846
Hyp 3c: Type IIa-1 and IIa-3 subfamilies are monophyletic to the exclusion of all other clades	Positive	2.000**	0.008	0.127
Hyp 4: Type IIa-3 and IIb subfamilies are monophyletic to the exclusion of all other clades	Positive	4.070 *	> 0.001	> 0.001
Hyp 5: Tunicate and vertebrate receptors are monophyletic to the exclusion of all other clades	Positive	30.900**	0.624	0.968

Hypotheses tested using topological constraint tests, which are also illustrated in Figure 7. Hypotheses were compared to the null hypothesis that vertebrate receptors are monophyletic. Specified clades for each test were either constrained to be monophyletic (positive constraint) or not allowed to be monophyletic (negative constraint; limited to Bayesian analyses). Significant results indicate rejection of the hypothesis if the values are positive and acceptance if the values are negative. Bayes Factor scores result from comparisons to a null model with a positive constraint on the vertebrate branch, using a stepping stone model [37]. The strength of the inference is * = strong or ** = very strong [40]; AU = *p*-values for the Approximately Unbiased test [38]; SH = *p*-values for the Shimodaira-Hasegawa test [39].

## Discussion

### Three GnRH receptors were isolated from axolotls

We isolated three GnRH receptor genes from axolotls (Figures 1, 2, 3 and 4), which appear to be orthologs of the three genes that have been reported in frogs [10]. The naming conventions for GnRH receptor homologs have been based on the order of discovery, putative homology to the mammalian Type I GnRH receptor, expression in the pituitary, or the structure of the third extracellular loop e.g., [10,23,41]. We chose to follow the convention introduced by Roch et al. [14] because it is based on the evolutionary relationships among genes. Thus, we have designated the three axolotl GnRH receptors as Type IIa-2, IIa-3, and IIb, which are orthologous to bullfrog genes that had been named type 1, 3, and 2 GnRH receptors, respectively (Figure 5).

The evolutionary relationships among axolotl GnRH receptor genes relative to their homologs from both vertebrates and invertebrates were determined by phylogenetic analyses. As illustrated in Figure 5, the results indicate that vertebrate GnRH receptor genes form a strongly supported monophyletic clade that excludes all invertebrate homologs, including some that had previously been identified as GnRH receptors. Our results differ from those of other recent evolutionary analyses of GnRH receptors, including that of Roch et al. [14], who used maximum likelihood phylogenetic methods based on sequences of GnRH receptors to infer phylogenies, and that of Kim et al. [13] and Sefideh et al. [15], who analyzed syntenic relationships to estimate evolutionary relationships among the vertebrate GnRH receptor genes. In addition to differences in taxonomic coverage, our analyses differ in that we used both maximum likelihood and Bayesian methods, which can outperform likelihood-based approaches in reconstructing phylogenetic histories, particularly with respect to gene family-based analyses [42-45]. We also tested among explicit alternative phylogenetic hypotheses with constrained topology tests, and the results provide robust support for the evolutionary scenarios we propose. Finally, we supplemented our phylogenetic analyses with probabilistic search methods that provide statistical support for the identification and orthology delineation for protein domains. The latter methods improved our ability to identify homologs from genome assemblies where full-length genes and gene annotations are absent.

### The GnRH receptors are related to receptors for Corazonin, AHK, and ACP

As suggested by Roch et al. [14], the GnRH receptor genes appear to be part of a larger superfamily that includes receptors for corazonin, AKH, and ACP, a novel peptide that resembles both corazonin and AKH but selectively activates a unique group of receptors [46]. These four subfamilies of receptors are distributed among clades of insects, mollusks, worms, and amphioxus, and are predominantly involved in reproduction, metabolism, and cardiac regulation. Evolutionary relationships among these receptor subfamilies are unresolved and multiple subfamily members are sometimes found within a species. Previous functional categorization has occasionally relied on sequence homology, but receptor-ligand affinity can be context dependent with receptors activated by multiple classes of hormones from heterologous taxa. The precise function of each GnRH receptor homolog from basal animal lineages will require biochemical or physiological data obtained in a biologically relevant, *in vivo* context.

Our results indicate that four of the putative GnRH receptor sequences that we found using BLAST searches of the amphioxus genome are closely related to the vertebrate GnRH receptors. These likely are functional GnRH receptors, as they bear substantial similarity to the amphioxus ‘receptors 1 and 2’ cloned by Tello and Sherwood, which they demonstrated can be activated by vertebrate GnRH 1 and 2 [47]. On the other hand, we found three additional receptor sequences (E-G) that cluster with the insect corazonin receptors, which are similar to Tello and Sherwood’s Type 3 and 4 receptors. In an inositol triphosphate (IP_3_) accumulation assay, the amphioxus Type 4 receptor did not respond to GnRH [47]. Interestingly, the Type 3 receptor was activated by AKH and by octopus GnRH, a dodecapeptide, rather than by vertebrate GnRHs [47], indicating that these amphioxus receptors have a function somewhat different from that of typical vertebrate GnRH receptors. We found further evidence of additional GnRH receptors in the amphioxus genome, but they were either nearly identical to the genes examined here or unique homologs that comprised partial gene sequences. This complex repertoire of vertebrate-like receptors (putative GnRH receptors) and insect-like receptors is unique among animals and further functional analyses will provide important clues towards elucidating the dynamic evolutionary history of GnRH receptor genes from amphioxus.

As illustrated in Figure 5, a clade containing receptors from tunicates (*Ciona intestinalis*) is basal to a clade containing the four amphioxus homologs. Three of the four tunicate receptors have been shown to respond to tunicate homologs of GnRH as well as to vertebrate GnRH2 [48], indicating that these are functional GnRH receptors. Nevertheless, in tunicates GnRH and GnRH receptors have functions beyond reproduction [49]. Tunicates are derived relative to amphioxus, so the ancestral position of the tunicate GnRH receptor genes is inconsistent with the evolutionary history among taxa. Roch et al. (2011) hypothesized that relatively rapid evolution of the tunicate receptors may explain their ancestral placement in the phylogeny, but the branch lengths observed in their study and ours do not indicate unusual rates of evolution for these genes. Bayes Factor constrained topology tests rejected the hypothesis that tunicate GnRH receptors are derived relative to amphioxus receptors A through D, although the Maximum Likelihood tests failed to reject the hypothesis (Table 2). In summary, consensus trees, bootstrap support, and posterior probabilities support a amphioxus clade derived relative to tunicates, but explicit hypothesis tests provided inconsistent results; thus, the relative positions of tunicates and amphioxus GnRH receptor genes is not clear.

Three putative receptors that we obtained through BLAST searches of the sea urchin (*Strongylocentrotus purpuratus*) genome have also been classified as GnRH receptors based on sequence similarity [14]. As with the tunicate receptors our analyses indicate that the three sequences form a monophyletic clade, likely due to lineage-specific gene duplication. Nevertheless, the sea urchin GnRH receptor clade was consistently ancestral to both tunicates and the amphioxus GnRH receptors A through D. Because the phylogenetic position of the clades that contain AKH receptors and ACP receptors is uncertain with respect to both tunicate and sea urchin genes, determination of the precise function of the sea urchin genes will require functional assays. These results serve as a reminder to exercise caution in equating sequence similarity with ligand selectivity. Finally, phylogenetic analyses failed to resolve the topological position of the clades containing the AKH, ACP, and CRZR receptors (Figure 5). Resolution of these clades will be important in understanding whether regulating reproduction is the original function of these receptors or is an evolutionary novelty gained in the GnRH receptors.

### Vertebrates possess five subfamilies of GnRH receptors

Our analysis revealed the presence of five subfamilies of GnRH receptors in vertebrates; remarkably, coelacanths possess intact genes for all five subfamilies. Roch et al. [14] found phylogenetic support for three large subfamilies of GnRH receptors and we followed their nomenclature in naming the Type I, Type IIa, and Type IIb subfamilies of GnRH receptors. Several additional analyses are concordant with monophyly for clades that we label Type I, IIa-2, IIa-3, and IIb. The clade that Kim and colleagues [13] call mammalian Type I (GnRHRm1) is the same as our Type I, their clade nonmammalian Type I (GnRHRn1) is equivalent to our Type IIa-2, their nonmammalian Type III/ mammalian Type II (GnRHRn3/m2) is equivalent to our Type IIa-3, and their nonmammalian Type II (GnRHRn2) is equivalent to our Type IIb. Using a neighbor-joining algorithm, Chen and Fernald [12] described four subfamilies, which they designated a1 (equivalent to our Type IIb), a2 (equivalent to our Type I), b1 (equivalent to our Type IIa-3), and b2 (equivalent to our Type IIa-2). Recent synteny analyses by Sefideh and colleagues [15] resulted in classification of six extant paralogs that they interpret as remnants of eight ancestral paralogs, which arose through a pair of gene duplicates undergoing two rounds of genome duplication. Thus, the relationships among these groups of receptors are controversial: our results suggest a different interpretation than those that have been outlined by previous researchers.

**Figure 8:**
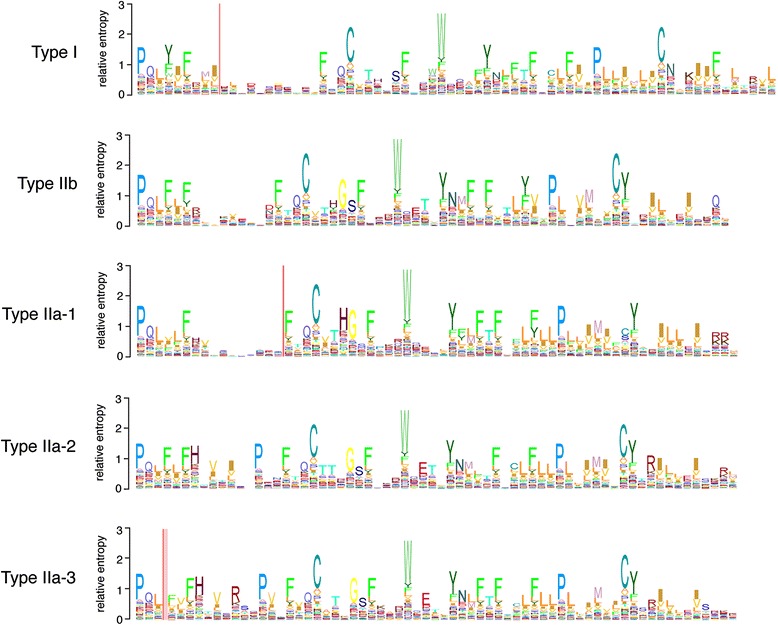
**Logos for representative HMM profiles from each of the five subfamilies of GnRH receptors.** The physical location of the region used to construct this HMM model, named ‘TM4 to 5′ in Additional file 3: Table S3 roughly corresponds with the C-terminal half of trans-membrane domain 4 to the C-terminus of transmembrane domain 5 (Figure 10). The relative entropy score for each position in the HMM profile is indicated by the height of stacked single-letter abbreviations for amino acids. Vertical red lines indicate the presence of an indel and pink shading to the right of the line represents an estimate of size variation for each respective indel. Logos were generated using LogoMat-M [50].

Although our analysis and that of Roch et al. [14] agree with respect to the assignment of homologs within subfamilies, our results differ in the number of homologs identified, the number of subfamilies named and the order of subfamily origin. We further subdivided the Type IIa subfamily into Type IIa-1, IIa-2, and IIa-3. Although Roch et al. [14] did not formally distinguish between the Type IIa-2 and Type IIa-3 subfamilies, both clades were strongly supported in their analyses. Our decision to further subdivide the Type IIa subfamily is based on strong branch support from phylogenetic analyses with results robust to methods of phylogenetic inference, models of evolution (Figure 5 and Additional file 5: Figure S1), and decreased taxon sampling (Additional file 6: Figure S2). In addition, constrained topology tests reject hypotheses that prohibit monophyletic Type IIa-1, IIa-2, or IIa-3 subfamilies (Table 2 and Figure 7). Finally, HMMER search results consistently distinguish among the GnRH receptor subfamilies with strong statistical support for subfamily orthology, in which search results were derived using every HMM domain and every putative homolog as queries in multiple searches. Visual inspection of an exemplar HMM ‘logo’ , which provides a graphical depiction of an HMM profile, highlights several unique sequence motifs that characterize differences among the GnRH receptor subfamilies (Figure 8).

In a recent paper, Sefideh and colleagues [15] note the presence of a mammalian Type I receptor in the coelacanth as well as an additional coelacanth receptor ‘2c’ , which is equivalent to our Type IIa-1 subfamily. However, their evolutionary analyses are largely based on syntenic gene order in teleosts, coelacanths, *Xenopus*, chickens, and humans. They propose a model in which an ancestral pair of physically linked gene duplicates underwent two rounds of genome duplication resulting in eight genes, or four pairs of physically linked gene duplicates. Subsequent gene loss, translocation, chromosome fusion, and losses of large chromosome segments are hypothesized to explain the current distribution of paralogs. Their nomenclature separates subfamilies into two groups, one for each gene in the putative ancestral pair of linked duplicate genes. The first gene in the pair gave rise to 1a (equivalent to our Type I) and to 1b and 1c (equivalent to our Type IIb) and the second gene gave rise to 2a, 2b, and 2c (equivalent to our Type IIa-1, IIa-2 and IIa-3, respectively). In addition, our interpretation differs from that of Sefideh et al. in that they propose reciprocal gene loss for two pairs of paralogs with the result of one paralog unique to sarcopterygians (their 1b) and another unique to actinopterygians (their 1c); in constrast, we hypothesize the existence of a single ortholog (our Type IIb).

Sefideh et al. used phylogenetic analyses to classify their six subfamilies, but nearly all branches on their phylogeny exhibit low bootstrap support [15]. In addition, their phylogenetic results are described as two deeply divided clades resulting from the ancestral pair of physically linked gene duplicates, but those clades are poorly supported and the remainder of the phylogeny comprises polyphyletic subfamilies of GnRH receptors. Syntenic patterns also fail to support their hypothesized model. The syntenic pattern among many genes demonstrates three blocks of syntenic paralogs; however, GnRH receptor duplication events appear to have been mapped onto the ancestral chromosomes in an *ad hoc* manner. For example, the type 1c gene is indicated at a syntenic location on figures despite its absence in tetrapods, and the type 1b gene is indicated as an absent syntenic gene in all teleosts. Further, they note that a fourth pair of proximate duplicate genes is missing, because they found six subfamilies instead of the eight that would be expected from the two rounds of whole-genome duplication that occurred in early vertebrates. Finally, they also note non-syntenic placement of subfamilies 1a and 2a. The authors explain this unexpected pattern through a gene loss from one pair of duplicates (loss of 1a’), followed by a translocation event that replaced the lost gene (1a moved to replace 1a’), followed by another gene loss (loss of 2a’).

In contrast to the model proposed by Sefideh et al. [15], we propose that their subfamilies named 1a and 2a are not syntenic, while subfamilies 1b and 1c are a single family. This results in five subfamilies instead of their proposed six, with only one pair of syntenically proximate subfamilies, Type IIa-2 and IIb. Finally, all of the evolutionary events proposed in their model, including the original duplication, two rounds of genome duplication and the reciprocal gene loss plus translocation, are presumed to have transpired during the short time span that separated the common ancestor of actinopterygians from the common ancestor of sarcopterygians. In summary, while we agree with their identification of a Type I receptor, we disagree with respect to the number of subfamilies, the order of subfamily origins, the mechanisms behind subfamily origins, and the timing of duplication events.

### Relationships among the five subfamilies of GnRH receptors

**Figure 9:**
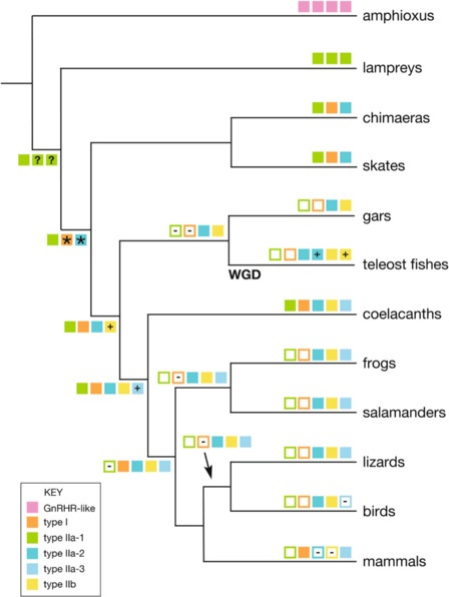
**Summary of the hypothesized evolutionary history of the GnRH receptor family in vertebrates.** Boxes and their respective colors represent major groups of paralogous gene family members, as illustrated in Figure 5. Filled boxes symbolize gene presence and open boxes symbolize gene absence. “-” in an open box indicates the initial loss of a gene and “+” indicates the initial gain of a gene. “?” indicates that insufficient data are available to determine whether the ancestral condition is presence of one, two, or three copies of a Type IIa-3 receptor, and “*” indicates receptors that arose either through duplication or divergence. “WGD” signifies the third lineage-specific whole genome duplication that occurred in teleosts. Amphioxus possess four copies of a gene that is closely related to the GnRH receptors, as illustrated in Figure 5.

Phylogenetic analyses, such as that depicted in Figure 5, may be misleading with respect to the relative placement of subfamilies due to unequal rates of sequence evolution and poor taxonomic sampling. The combination of these factors can result in excess homoplasy, termed long branch attraction [42], which results in strong but misleading phylogenetic support for some branches on a tree. Therefore, rather than relying solely on such analyses, we base our inferences on the identification of novel homologs via HMMER searches combined with phylogenetic results. We were able to classify homologs into their respective subfamilies with statistical significance using HMM models, which then facilitated mapping of orthologs onto the species tree shown in Figure 9. Using the combined results of these analyses, we propose a new hypothesis concerning the order of origin for GnRH receptor subfamilies that is the inverse of that proposed by previous researchers.

The Type IIa receptor subfamilies are ancestral among vertebrates, and the classification of Type IIa-1, IIa-2 and IIa3 subfamilies helps clarify their origins. Specifically, the earliest vertebrate GnRH receptors were members of the Type IIa-1 subfamily. Results from HMMER searches revealed orthologous Type IIa-1 receptors in the chimera, little skate, and coelacanth. In addition, statistical classification using HMMER indicate that all three lamprey genes are members of the Type IIa-1 subfamily (Table 1, Additional file 1: Table S1 and Additional file 2: Table S2).

Previous phylogenetic analyses have failed to identify the Type IIa-1 subfamily, which is likely due to the limited sample of three lamprey receptors resulting in weak support for the phylogenetic position of these genes [13,14]. Our results indicate a paraphyletic subfamily that comprises strong support for a pair of lamprey receptors monophyletic with a coelacanth receptor identified in this work as well as by Sefideh et al. [15], but ambiguous placement for the third lamprey gene. Constrained topology tests indicate that the phylogenetic position of the Type IIa-1, IIa-2, and IIa-3 subfamilies is unresolved. As a result, we focused on the taxonomic distribution of orthologs classified using HMMER searches to resolve the order of subfamily evolution.

Our data do not resolve the number of GnRH receptors in the common ancestor of vertebrates. The tunicate genes as well as those of amphioxus likely arose either through lineage specific duplications of a single ancestral gene or lineage specific gene conversion events among paralogous genes, resulting in two monophyletic clades in these two groups. The lamprey is the earliest-diverging vertebrate in our sample and it also harbors three receptors in one subfamily, Type IIa-1. Further, the timing of the two rounds of whole genome duplication in early vertebrates remains controversial [51-54] and therefore does not allow us to draw conclusions concerning the timing and nature of the duplications that gave rise to the GnRHR family in vertebrates. At present, the available data are consistent with at least two different hypotheses that could explain the pattern of evolution depicted in Figure 9. First, an ancestral single copy Type IIa-1 gene may have undergone multiple duplications in the lamprey lineage in conjunction with independent duplications in the common ancestor of jawed vertebrates. Relatively radical evolution in two of the paralogs in the ancestor of jawed vertebrates could then have resulted in the origin of the Type I and IIa-2 subfamilies. Second, three ancestral vertebrate receptors might have been retained in the lamprey, followed by relatively rapid sequence evolution in the common ancestor of jawed vertebrates to give rise to the Type I and IIa-2 subfamilies. Clarifying the timing of duplication events that gave rise to these subfamilies will require additional taxon sampling.

Based on the taxonomic distribution of the orthologs, we propose that the Type I and IIa-2 receptors are derived from Type IIa-1 receptors, as the Type IIa-1 subfamily was present before all other vertebrate subfamilies. As illustrated in Figure 9, the taxonomic distribution of receptors also leads us to propose that Type IIa-3 receptors were derived from the Type IIa-2 subfamily. Cloning efforts have failed to find Type IIa-3 receptors in teleost fishes, and our search results indicate that it is absent from the genomes of teleosts and gars; thus, the taxonomic distribution of Type IIa-3 is the most limited of all subfamilies. Further, given their taxonomic distribution, we propose that the Type IIa-2 and IIa-3 subfamilies are the most derived sister clades in the phylogeny, corroborating the idea that the Type IIa-3 subfamily arose relatively recently through gene duplication.

In addition, we propose that the Type IIb subfamily is derived from the Type IIa-2 subfamily, although at an older evolutionary time point. The Type I subfamily is comprised of genes that lack the C-terminus relative to all other subfamilies, and it seems unlikely that these shorter genes could give rise to longer genes with convergent C-terminus sequences in the Type IIa-1 and IIa-2 subfamilies. Given their taxonomic distributions (Figure 9), the Type IIa-3 subfamily could not be the progenitor for the Type IIb subfamily. Thus, only the Type IIa-1 and IIa-2 subfamilies remain as potential ancestors of the Type IIb subfamily. The Type IIa-1 subfamily could be the ancestral origin for Type IIb, particularly given that constrained topology tests did not reject a sister relationship between the two subfamilies (hypothesis 3a in Table 2 and Figure 7). However, the Type IIa-2 and IIb subfamilies are consistently found on the same chromosome, flanked by some syntenic genes, indicative of a localized segmental duplication [13,15]. We therefore propose that the Type IIa-2 subfamily was the progenitor and gave rise to the Type IIb subfamily through localized segmental duplication.

**Figure 10:**
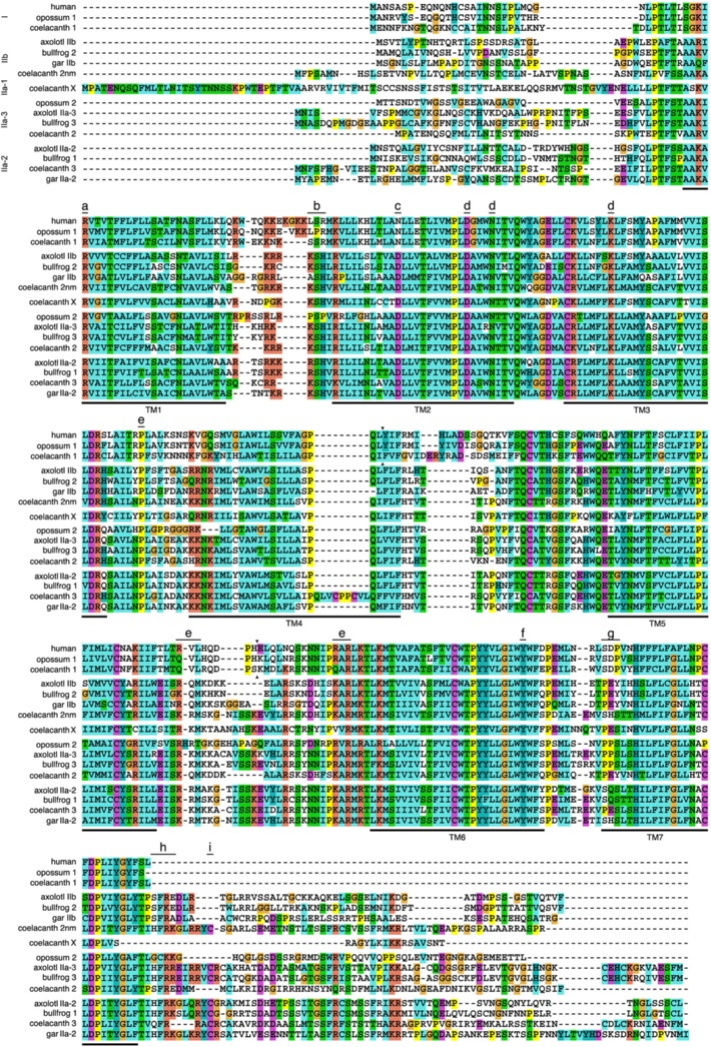
**Complete open reading frame sequences of representative GnRH receptors, aligned using Clustal X** **[** 58 **].** Arrowheads indicate intron-exon boundaries. Locations of transmembrane domains (TM) are estimated based on those illustrated in [25] and in [10]. **a** = GnRH binding site for Type I receptors [59]; **b** = G_s_ coupling site in Type I receptors [24]; **c** = GnRH2 binds receptors containing D at this position, and does not bind receptors containing an N at this position [56]; **d** = GnRH binding site in both Type I and II receptors [24,55]; **e** = G_q/11_ coupling site in Type I receptors [24]; **f** = GnRH binding site in Type II receptors [55]; **g** = sites contributing to differential sensitivity to GnRH1 and GnRH2 [57]; **h** = site involved in activation of the adenylyl cyclase/protein kinase A signaling pathway [60]; **i** = site involved in rapid internalization of Type II receptors [61].

The Type I receptors were previously known only from mammals and were thought to represent a recent adaptation within this group. Nevertheless, we were able to find Type I receptors in the genomes of a chimaera, skate, and coelacanth, indicating that this receptor type is phylogenetically much older than previously understood. The Type I receptors lack a cytoplasmic tail, a feature that will be discussed in more detail below. Many GnRH binding sites are highly conserved and are known to function as such in both Type I and II receptors e.g., [24,55], but some are known to confer differential sensitivity to the GnRH1 and 2. Specifically, the second transmembrane domain of Type II receptors contains a D residue that is critical for binding GnRH2, and the N at this site in the Type I receptors eliminates this binding (indicated with a “c” on Figure 10) [56]. The Type I receptors contain a short sequence (SDP or SEP) between the sixth and seventh transmembrane domains that is involved in binding GnRH1 (indicated with a “g” on Figure 10). This tripeptide sequence is present in coelacanths and heterologous expression assays indicate that, like the Type I receptor in mammals, the Type I receptor in coelacanths has a much higher affinity for GnRH 1 than for GnRH 2 or 3 [15]. In the chimaera these three amino acids are PEP; interestingly, Wang and colleagues [57] made exactly this mutation in a study of receptor-ligand specificity, and found that receptors bearing the sequence PEP at these sites have a higher affinity for GnRH2 than for GnRH1.

### The vertebrate GnRH receptor genes have been duplicated and lost repeatedly

Given the available data, the history of the vertebrate GnRH receptor subtypes cannot be determined with absolute certainty; nevertheless, solid inferences were used to propose the evolutionary hypothesis depicted in Figure 9.

Our BLAST searches of the genome of the spotted gar (*Lepisosteus oculatus*) [62], a ray-finned fish the ancestors of which diverged before the teleost specific genome duplication event, revealed the presence of single copy Type IIa-3 and IIb receptor genes. Thus, both subtypes must have arisen at or before the origin of actinopterygians. In addition, a common ancestor of gars and teleosts appears to have lost the Type I and IIa-1 receptor genes. Teleosts generally possess two copies of the Type IIa-2 and IIb receptor genes, a result consistent with a third round of whole-genome duplication that occurred in this lineage [63]. Additional duplications have occurred in some lineages; for example, pufferfish (*Tetraodon nigroviridis*) possess three copies of the Type IIa-3 receptor.

Amphibians have lost the Type I GnRH receptor. Cloning efforts in other labs and our own have failed to find a homolog of the Type I receptor in axolotls or frogs [10,41]. Further, synteny analysis indicates that the gene is missing in *Xenopus tropicalis* [13]. The gene is also absent in anoles, zebra finches, and chickens [13], suggesting that it was lost independently in reptiles.

The Type IIa-3 receptor may have been lost in birds. Specifically, we were unable to find Type IIa-3 GnRH receptor genes through BLAST searches of the genome of the domestic chicken, *Gallus gallus domesticus* [64], domestic turkey, *Meleagris gallopavo* [65], and zebra finch, *Taeniopygia guttata* [66]. Further, our syntenic analysis indicated that the gene is not present at the syntenic position in zebra finch. On the other hand, the gene appears to be present in other groups of reptiles. This receptor subtype was cloned from the common leopard gecko, *Eublepharis macularius* [67], and our syntenic analysis indicates that it is also present in the western painted turtle, *Chrysemys picta bellii* [68] and the Chinese softshell turtle, *Pelodiscus sinensis*. Until additional taxon sampling can fully resolve the timing of gene loss, we can conclude that the Type IIa-3 subfamily appears to have been lost in the common ancestor to birds. Whether additional reptiles or crocodilians have also lost the gene remains unresolved.

All mammals examined to date possess Type I GnRH receptors; some also possess the Type IIa-3 receptor, although the latter has been lost independently several times. As illustrated in Figure 5, the Type IIa-3 receptor gene is present in vervet monkeys (*Chlorocebus aethiops*), rhesus monkeys (*Macaca mulatta*), bonnet macaques (*Macaca radiata*), and opossums (*Monodelphis domestica*). It is present but unannotated in the genome of the platypus (*Ornithorhynchus anatinus*) and is a pseudogene in humans [13]. In fact, the reason that the repertoire of GnRH receptors in humans is unique is not due to the origin of a novel form of receptor. Instead humans should be viewed as among the most degenerate species with respect the GnRH receptor evolution because we retained only one member of five potential subfamilies, and the retained gene is from one of the most ancient subfamilies. This result suggests that some types of GnRH receptors can be lost readily, and that generalizations about broad taxonomic groups should be made cautiously.

### The Type I GnRH receptor is not unique to mammals

The Type I GnRH receptor is unusual in that it lacks a cytoplasmic tail at the C terminal. More commonly, G-protein coupled receptors possess a long cytoplasmic tail that contains multiple phosphorylation sites. Ligand binding activates the receptor, stimulating a second messenger cascade that results in phosphorylation of these sites, which both desensitizes the receptor by preventing G-protein coupling and facilitates internalization of the receptor-ligand complex through β-arrestin binding [e.g., 61]. The Type II GnRH receptors possess a typical cytoplasmic tail, desensitizing and internalizing within minutes of ligand binding [69-71]. By contrast, the Type I receptor desensitizes and internalizes over the course of hours, a process that involves downstream elements, including downregulation of IP_3_ and reduced mobilization of intracellular calcium [8,22,72,73]. The functional significance of this slow desensitization and internalization is unknown, but has been assumed to reflect a specific adaptation to the reproductive biology of mammals.

Nevertheless, our analysis indicates that the Type I receptor is much older than previously thought, as we found copies in the genomes of a skate, chimaera, and coelacanth, the latter of which was also noted by Sefideh and colleagues [15]. Chimaeras clearly possess this gene: we found the three exons on separate contigs in the elephant shark genome, and Ikemoto and Park cloned identical cDNA from this species (unpublished; GenBank accession number ABU55292). Although the skate homolog was identified only as a partial sequence, the putative Type I receptors in chimaeras and coelacanths are full-length sequences that lack a cytoplasmic tail. To determine whether gene length alone was responsible for the phylogenetic affinity among putative Type I receptors, we deleted the amino acids beyond the seventh transmembrane domain from all Type II receptor sequences. The putative Type I receptor sequences from chimaera and coelacanth still clustered with those known to be Type I receptors in mammals, demonstrating that the lack of a cytoplasmic tail did not skew the results of our analysis. Thus, we are confident in assigning these genes from chimaeras and coelacanths to the Type I receptor clade.

The absence of the cytoplasmic tail in the Type I receptor is puzzling. Given its phylogenetic distribution, the slow receptor internalization cannot be an adaptation to mammalian physiology. Early researchers who discovered the slow internalization kinetics of the Type I GnRH receptor speculated that this characteristic might be required for accurate temporal resolution of the GnRH pulses that cause the preovulatory surge of luteinizing hormone (LH) in mammals [e.g., 23,72]. However, preovulatory LH surges also occur in birds [74], which possess rapidly desensitizing pituitary GnRH receptors [19]. In addition, bullfrogs lack the Type I GnRH receptor, and all three GnRH receptor subtypes in these animals desensitize rapidly [75]. Nevertheless, exposure to GnRH pulses leads to desensitization in frogs [76], and prolonged exposure to GnRH leads to an LH surge [76-78]. Thus, the relationships among receptor desensitization/internalization, GnRH pulses, and gonadotropin release differ considerably across vertebrates.

## Conclusion

GnRH receptors have been duplicated and lost many times independently across the vertebrate lineage; the earliest vertebrates may have possessed a single GnRH receptor. Vertebrate GnRH receptors can be categorized into five subfamilies and the order of subfamily evolution is the inverse of previous hypotheses. One of these subfamilies, previously known only from the mammalian pituitary, dates back at least to the origin of cartilaginous fishes, calling into question the functional significance of the slow internalization dynamics of this receptor subtype.

## Methods

### Animals

Adult axolotls (*Ambystoma mexicanum*) of both sexes were used in all experiments. Axolotls were obtained from the Ambystoma Genetic Stock Center (University of Kentucky) and maintained at 20°C in Holtfreter’s solution, which contains (in mM): 60 NaCl, 2.4 NaHCO_3_, 0.67 KCl, 0.81 MgSO_4_, and 0.68 CaCl_2_ (pH 7.5 – 7.6). The light cycle in the colony was changed monthly to match that of the animals’ native habitat in Mexico City. Animals were fed commercial salmon pellets (Rangen, Buhl, ID) two or three times each week. All procedures were approved by and conducted under the supervision of the institutional animal care and use committee at Michigan State University (approval no. 11/06-130-00), in accordance with guidelines established by the US Public Health Service.

### RNA extraction and cDNA cloning

Total RNA from the brains of three adult axolotls was prepared using Isogen (Nippon Gene, Tokyo, Japan). A small amount of total RNA (1 μg) was reverse transcribed into DNA using 5 U of reverse transcriptase (MMLV; Promega, Madison, WI) in a final volume of 20 μl containing 1 x RT buffer (Promega), 1 mM of deoxyribonucleotide triphosphate, 10 ng/μl of random primer (Promega), and 20 U RNase inhibitor (Promega). The reaction was performed for 1 h at 42°C, followed by heat inactivation at 95°C for 10 min.

To amplify fragments of axolotl GnRH receptor cDNAs, degenerate PCR primers (GnRHR-SE1,2, and GnRHR-AS1,2,3; Additional file 4: Table S4) were designed based on conserved sequences of amphibian and teleost GnRH receptors. PCR was performed in a final volume of 10 μl containing 1x Taq polymerase buffer (Takara, Shiga, Japan), 200 μM of deoxyribonucleotide triphosphate, 0.5 U DNA polymerase (Taq polymerase, Takara), 1 μM degenerate primers, and 1 μl first-strand cDNA or the first-round PCR product. Reaction conditions for PCR were 94°C for 1 min; 20–35 cycles of 94°C for 30 sec, 55°C for 30 sec, and 72°C for 30–60 sec; and then 72°C for 5 min. PCR products were separated on 1.5% agarose gels, and cDNA fragments with proper length were sequenced using an ABI PRISM 3100 DNA sequencer (Applied Biosystems, Foster City, CA). Using the resultant sequence data, we further designed gene-specific primers for 5′ and 3′ rapid amplification of cDNA ends (RACE).

100 μg isolated total RNA was further purified to recover polyA + RNA using an Oligotex-dT Super kit (Takara). 1 μg of polyA + RNA was applied to the SMART RACE kit (Clontech Laboratories, Palo Alto, CA) according to the manufacturer’s protocol for generation of 5′ and 3′ adaptor-ligated first-strand cDNA synthesis. Primary amplification was performed using a universal primer mixture (UPM, Clontech/Takara), complimentary to the adaptor sequence provided with the kit and gene specific primers (listed in Additional file 4: Table S4). PCR was performed in a final volume of 10 μl containing 1x Taq polymerase buffer (Takara), 250 μM deoxyribonucleotide triphosphate, 0.5 U DNA polymerase (Taq polymerase, Takara), 1 μM degenerated primers, 1 μl first-strand cDNA, and 10 mM of dithiothreitol. PCR conditions were as follows: 94°C for 5 min; 5 cycles at 94°C for 30 sec and 72°C for 2.5 min; 5 cycles at 94°C for 30 sec, 70°C for 30 sec, 72°C for 2 min; and 18 cycles at 94°C for 30 sec, 64°C (for R1 and R3) or 55°C (for R2) for 30 sec and 72°C for 2 min. Secondary or nested PCR was performed using 0.2 μl of the primary PCR product, a nested universal primer (NUP) complimentary to the adaptor sequence provided with the kit, and a gene-specific nested primer. PCR conditions were as follows: 94°C for 5 min; 5 cycles at 94°C for 30 sec and 72°C for 2.5 min; 5 cycles at 94°C for 30 sec and 70°C for 30 sec, 72°C for 2 min; and 25–30 cycles at 94°C for 30 sec, 64°C (R1 and R3) or 55°C (R2) for 30 sec and 72°C for 2 min. The PCR products were gel purified and some were ligated into the pGEM-T plasmid vector (Promega). Sequencing was performed using an ABI Prism 3100 Sequencer or by Fasmac Co., Ltd. (Tokyo, Japan), using primers listed in Additional file 4: Table S4. Three independent positive clones from distinct amplifications were sequenced to avoid PCR error.

### Phylogenetic analysis of GnRH receptors

Vertebrate homologues of the GnRH receptors were identified through a combination of reciprocal BLASTp, TBLASTn, and TBLASTx searches using axolotl and human GnRH receptors as the query sequences in searches among well-annotated genomes in the NCBI and ENSEMBL databases. Additional homologs sequenced through cloning efforts were included for phylogenetically informative taxa. Finally, to obtain a better picture of outgroup and ancestral sequences, BLAST searches of some invertebrate genomes were conducted; our selection of species to be examined was guided by the data presented by Roch and colleagues [14]. In all cases, only full length GnRH receptor sequences were included in the analyses, and partial sequences and splice variants were excluded.

We also used a complementary approach of Hidden Markov Model (HMM) domain searching to identify homologs in the elephant shark, skate, lamprey and gar genomes. We focused the HMM searches on these species because these genome assemblies exhibited small contigs, which meant that sequence representation for full length genes was unlikely. In addition, each of these species holds a phylogenetically informative position with respect to many of the hypotheses addressed here.

Results from BLAST searches of the elephant shark and little skate genomes indicated that at least two GnRH receptor homologs are present, but sequence matches for each homolog were short in length and located on two separate contigs; *i.e.*, four total contigs with two contigs per homolog and two homologs per species. We suspect each of the four sequences correspond to exons, which is consistent with the large number of small exons found in many GnRH receptor genes. The physical locations of the sequence matches along the canonical GnRH receptor gene are indicated in Table 1 and Additional file 3: Table S3 and in Figure 10. Each of the four BLAST matches was used to define physical landmarks along the GnRH receptor genes. Next, all homolog protein sequences were manually trimmed at established landmarks to create four different sequence fragments that were then used to generate HMM profiles using HMMER v. 3.1 (named TM1-4, TM4-5, TM6 and TM6-7 in Table 1, Additional file 2: Table S2 and Additional file 3: Table S3 and Figure 10) [34]. Construction of the HMM profiles was further subdivided among GnRH receptor subclades (Figure 5) by limiting sequence inputs to only those sequences from each subclade.

HMM analyses were carried out using the elephant shark genome assembly version 1.4, the little skate assembly ‘build 2’ , lamprey genome assembly version 7.0 and the spotted gar genome assembly version 1.0. Each DNA genome database was translated in all six frames using the ‘transeq’ tool from EMBOSS v. 6.4.0. Translated genome databases were searched with HMM profile queries using hmmsearch, from HMMER v. 3.1. Each HMM profile was generated using the hmmbuild program and profiles were indexed for searches using the hmmpress program, both from HMMER v. 3.1. In order to cross validate each HMM-profile-to-sequence match, we used each sequence as a query to search all HMM profiles from the Pfam database (version available on November 20, 2013), including all HMM profiles generated in this work.

Sequences were aligned using the MUSCLE algorithm with default settings as implemented in Mega 5.2 [79]. RaXML v.7.7.1 [80] was used to estimate the maximum likelihood tree from 10 independent, random starting tree topologies and 100 bootstrap pseudoreplicates. Likelihood values were calculated using a JTT substitution matrix, empirical amino acid frequencies, with both the parameters for the shape of the gamma distribution of rate variation and the proportion of invariant sites estimated from the data (Additional file 5: Figure S1). The software package Mr. Bayes [81] was used to construct Bayesian inference trees, with two independent runs of 4,000,000 generations among four chains with model averaging for amino acid sequence data. Trees were saved every 500 generations and the first 25% of generations were discarded as burnin. Stationarity over the Markov chain Monte Carlo generations was determined based on several observations: both runs produced identical topologies, the average standard deviation of the split frequencies (ASDSF) fell below the suggested value of 0.01 after 500,000 generations and reached a final value of 0.005, the potential scale reduction factor (PSRF) score was 1.0 ± 0.001 for all parameter estimates, and there was no pattern among likelihood values plotted across generations. Patterns of syntenic relationships among genes were determined by manual inspection of genes localized on contigs as well as analyses using the Genomicus genome browser [82].

Hypotheses concerning the topological ordering and monophyly of GnRH receptor subclades were tested using three approaches. First, constrained topology searches and SH tests were carried out using RAxML v. 7.7.8, which was limited to hypotheses that require positive constraints (Table 2). Second, approximately unbiased tests were carried out using CONSEL, with input topologies generated from the constrained searches in RAxML as described in the previous step. Third, a stepping stone model for Bayes Factor topology testing was applied to positive and negative topological constraints (Table 2) using MrBayes v. 3.2.1, following the recommendations for topology constraints on null hypotheses as described in Bergsten et al. [37].

## Additional files

Additional file 1: Table S1. Sequences referenced in the text and in Figures 4, 5 and 6. Additional file 2: Table S2. Skate and lamprey gene homology determined with putative homolog sequences searched against HMM profiles using HMMER. Additional file 3: Table S3. Gar and chimaera gene homology determined with GnRH receptor type-specific HMM profiles using HMMER. Additional file 4: Table S4. Nucleotide sequences of primers used for PCR amplification of GnRH receptor genes in axolotls. Additional file 5: Figure S1. Maximum likelihood tree illustrating the evolutionary relationships among GnRH receptor homologs from animals, generated using the program RaXML [77] and rooted with the human oxytocin and vasopressin receptors. Numbers above branches indicate bootstrap support values. Two lancelet sequences, A and B, differ by a single amino acid; thus, only one was included in the analysis. ACPR = adipokinetic hormone/corazonin-related peptide receptor; AKHR = adipokinetic hormone receptor; CRZR = corazonin receptor; OTR = oxytocin receptor; V1bR = Type 1b vasopressin receptor. Additional file 6: Figure S2. Cladograms from Bayesian analyses depicting the evolutionary relationships among the genes encoding receptors for GnRH and other peptides; omitting potentially problematic sequences does not substantially alter the topology relative to that shown in Figure 5. Colored backgrounds emphasize strongly-supported, monophyletic subfamilies of GnRH receptors as shown in Figure 5. Numbers indicate the posterior probability support value for the corresponding branch located to the left of the value. Latin names of species and accession numbers for sequences are provided in Additional file 1: Table S1. (a) Tree containing only putative GnRHR sequences from chordates, with all basal taxa removed. (b) As in (a), but also omitting sequences from lampreys. (c) As in (b), but also omitting the coelacanth X sequence.

## Abbreviations

ACPR: Adipokinetic hormone/corazonin-related peptide receptor
AKHR: Adipokinetic hormone receptor
CRZR: Corazonin receptor
GnRH: Gonadotropin releasing hormone
OTR: Oxytocin receptor
V1bR: Type 1b vasopressin receptor
