# Supplementary material for: Dynamic evolution of the GnRH receptor gene family in vertebrates
Source: BMC Evol Biol. 2014 Oct 25;14:215. doi: 10.1186/s12862-014-0215-y (PMC4232701; doi:10.1186/s12862-014-0215-y)
Supplement: Additional file 1: Table S1. — Sequences referenced in the text and in Figures 4, 5 and 6. [file 12862_2014_215_MOESM1_ESM.docx]

**Supplemental Table 1:** Sequences referenced in the text and in Figures 2 - 4.

| **name used** | **species** | **database** | **accession no.** |
| --- | --- | --- | --- |
| axolotl IIa-2 | *Ambystoma mexicanum* | GenBank | KF499141 |
| axolotl IIa-3 | *Ambystoma mexicanum* | GenBank | KF499142 |
| axolotl IIb | *Ambystoma mexicanum* | GenBank | KF499143 |
| bee CZR | *Apis mellifera* | GenBank | NP_001137393 |
| bee AKHR | *Apis mellifera* | GenBank | NP_001035354 |
| buffalo | *Bubalus bubalis* | GenBank | ABG88199 |
| bullfrog 1 | *Rana catesbeiana* | GenBank | AAG42575 |
| bullfrog 2 | *Rana catesbeiana* | GenBank | AAG42949 |
| bullfrog 3 | *Rana catesbeiana* | GenBank | AAG42574 |
| caecilian | *Typhlonectes natans* | GenBank | AAD49750 |
| chicken 1 | *Gallus gallus* | GenBank | NP_989984 |
| chicken 2 | *Gallus gallus* | GenBank | NP_001012627 |
| chimaera | *Callorhinchus milii* | GenBank | ABU55292 |
| clawed frog 1 | *Xenopus tropicalis* | GenBank | ABO77122 |
| clawed frog 2 | *Xenopus tropicalis* | GenBank | ABO77123 |
| clawed frog 3 | *Xenopus tropicalis* | GenBank | ABO77124 |
| coelacanth 1 | *Latimeria chalumnae* | GenBank | KC478553 |
| coelacanth X | *Latimeria chalumnae* | GenBank | KC478554 |
| coelacanth 2 | *Latimeria chalumnae* | GenBank | KC478555 |
| coelacanth 2nm | *Latimeria chalumnae* | GenBank | KC478556 |
| coelacanth 3 | *Latimeria chalumnae* | GenBank | KC478557 |
| cow | *Bos taurus* | GenBank | AAC48857 |
| dog | *Canis lupus familiaris* | GenBank | AAF87097 |
| fruit fly CZR | *Drosophila melanogaster* | GenBank | NP_648571 |
| fruit fly AKHR | *Drosophila melanogaster* | GenBank | NP_477387 |
| gar IIa-2 | *Lepisosteus oculatus* | GenBank | AHAT01028092 |
| gar IIb | *Lepisosteus oculatus* | GenBank | AHAT01005053 |
| gecko 1 | *Eublepharis macularius* | GenBank | ABB89900 |
| gecko 2 | *Eublepharis macularius* | GenBank | BAD11150 |
| gecko 3 | *Eublepharis macularius* | GenBank | ABB89901 |
| goat | *Capra hircus* | GenBank | ABL76162 |
| gourami 1-1 | *Colisa lalia* | GenBank | BAE87048 |
| gourami 1-2 | *Colisa lalia* | GenBank | BAE87049 |
| gourami 2-1 | *Colisa lalia* | GenBank | BAE87050 |
| guinea pig | *Cavia porcellus* | GenBank | AAN75711 |
| horse | *Equus caballus* | GenBank | NP_001075305 |
| human | *Homo sapiens* | GenBank | AAA35917 |
| human OTR | *Homo sapiens* | GenBank | NP_000907 |
| human V1bR | *Homo sapiens* | GenBank | 2102273A |
| lamprey 1 | *Petromyzon marinus* | GenBank | AAQ04564 |
| lamprey 2 | *Petromyzon marinus* | GenBank | ABO77118 |
| lamprey 3 | *Petromyzon marinus* | GenBank | ABO77119 |
| amphioxus A | *Branchiostoma floridae* | JGI Genome Portal | fgenesh2_pg.scaffold_15000066 |
| amphioxus B | *Branchiostoma floridae* | JGI Genome Portal | fgenesh2_pg.scaffold_216000025 |
| amphioxus C | *Branchiostoma floridae* | JGI Genome Portal | fgenesh2_pg.scaffold_216000027 |
| amphioxus D | *Branchiostoma floridae* | JGI Genome Portal | fgenesh2_pg.scaffold_15000064 |
| amphioxus E | *Branchiostoma floridae* | JGI Genome Portal | fgenesh2_pg.scaffold_15000102 |
| amphioxus F | *Branchiostoma floridae* | JGI Genome Portal | e_gw.629.3.1 |
| amphioxus G | *Branchiostoma floridae* | JGI Genome Portal | e_gw.541.24.1 |
| amphioxus H | *Branchiostoma floridae* | JGI Genome Portal | gw.87.75.1 |
| macaque | *Macaca radiata* | GenBank | AAG43378 |
| marmoset 1 | *Callithrix jacchus* | GenBank | XM_002745789 |
| marmoset 2 | *Callithrix jacchus* | GenBank | XM_002759813 |
| marsh frog 1 | *Rana ridibunda* | GenBank | AAP15162 |
| marsh frog 2 | *Rana ridibunda* | GenBank | AAP15163 |
| marsh frog 3 | *Rana ridibunda* | GenBank | AAP15164 |
| medaka 1 | *Oryzias latipes* | GenBank | BAB70504 |
| medaka 2 | *Oryzias latipes* | GenBank | BAB70503 |
| medaka 3 | *Oryzias latipes* | GenBank | BAC97833 |
| mosquito ACPR | *Anopheles gambiae* | GenBank | ABX52399 |
| mouse | *Mus musculus* | GenBank | Q01776 |
| octopus | *Octopus vulgaris* | GenBank | BAE66647 |
| opossum 1 | *Monodelphis domestica* | GenBank | XP_001362289 |
| opossum 2 | *Monodelphis domestica* | GenBank | XP_001369168 |
| panda | *Ailuropoda melanoleuca* | GenBank | EFB14384 |
| pig 1 | *Sus scrofa* | GenBank | AAA31067 |
| pig 2 | *Sus scrofa* | GenBank | AAS68622 |
| platypus | *Ornithorhynchus anatinus* | GenBank | NP_001116830 |
| pufferfish 1-1 | *Tetraodon nigroviridis* | GenBank | BAE45695 |
| pufferfish 1-2 | *Tetraodon nigroviridis* | GenBank | BAE45697 |
| pufferfish 1-3 | *Tetraodon nigroviridis* | GenBank | BAE45699 |
| pufferfish 2-1 | *Tetraodon nigroviridis* | GenBank | BAE45701 |
| pufferfish 2-2 | *Tetraodon nigroviridis* | GenBank | BAE45703 |
| rabbit | *Oryctolagus cuniculus* | GenBank | NP_001076207 |
| rat | *Rattus norvegicus* | GenBank | AAC27349 |
| rhesus 1 | *Macaca mulatta* | GenBank | XP_001109227 |
| rhesus 2 | *Macaca mulatta* | GenBank | Q95JG1 |
| sheep | *Ovis aries* | GenBank | P32237 |
| silkworm ACPR | *Bombyx mori* | GenBank | NP_001127745 |
| tunicate 1 | *Ciona intestinalis* | GenBank | AAW70560 |
| tunicate 2 | *Ciona intestinalis* | GenBank | AAW70561 |
| tunicate 3 | *Ciona intestinalis* | GenBank | AAW70562 |
| tunicate 4 | *Ciona intestinalis* | GenBank | AAW70563 |
| urchin 1 | *Strongylocentrotus purpuratus* | GenBank | NP_001116990 |
| urchin 2 | *Strongylocentrotus purpuratus* | GenBank | NP_001116992 |
| urchin 3 | *Strongylocentrotus purpuratus* | GenBank | NP_001116991 |
| vervet | *Chlorocebus aethiops* | GenBank | AAK52746 |
| worm 1 | *Caenorhabditis briggsae* | GenBank | XP_002639368 |
| worm 2 | *Caenorhabditis briggsae* | GenBank | XP_002637834 |
| X. laevis 1 | *Xenopus laevis* | GenBank | AAF89754 |
| X. laevis 2 | *Xenopus laevis* | GenBank | AAK49334 |
| X. tropicalis 1 | *Xenopus tropicalis* | GenBank | ABO77122 |
| X. tropicalis 2 | *Xenopus tropicalis* | GenBank | ABO77123 |
| X. tropicalis 3 | *Xenopus tropicalis* | GenBank | ABO77124 |
| yak | *Bos grunniens* | GenBank | Q19PY9 |
| zebrafish 1 | *Danio rerio* | GenBank | ABU62656 |
| zebrafish 2 | *Danio rerio* | GenBank | ABU62657 |
| zebrafish 3 | *Danio rerio* | GenBank | ABU62658 |
| zebrafish 4 | *Danio rerio* | GenBank | ABU62659 |
