# Supplementary material for: Dynamic evolution of the GnRH receptor gene family in vertebrates
Source: BMC Evol Biol. 2014 Oct 25;14:215. doi: 10.1186/s12862-014-0215-y (PMC4232701; doi:10.1186/s12862-014-0215-y)
Supplement: Additional file 2: Table S2. — Skate and lamprey gene homology determined with putative homolog sequences searched against HMM profiles using HMMER. [file 12862_2014_215_MOESM2_ESM.docx]

**Table S2.** Skate and lamprey gene homology determined with putative homolog sequences searched against HMM profiles using HMMER.

|  | **Little Skate Genome** | | | | | |
| --- | --- | --- | --- | --- | --- | --- |
| Sequence ID | AESE011658775.1 | AESE012567234.1 | AESE011105720.1 | AESE010056425.1 | AESE011520245.1 | AESE011105720.1 |
| Domain Homology‡ | TM1 to TM4 | TM1 to TM4 | TM1 to TM4 | TM6 to TM7 | TM6 to TM7 | TM6 to TM7 |
| HMM-Type I | 2.8e-35 (113) | 3.6e-41 (132) | **9.2e-49 (156)** | 3.5e-27 (86) | 1e-35 (113) | **2.6e-35 (112)** |
| HMM-Type IIa-1 | **4.9e-57 (183)** | 6.2e-67 (215) | 1.4e-35 (114) | **1.9e-40 (128)** | 7.3e-40 (126) | 1.4e-28 (90) |
| HMM-Type IIa-2 | 1.1e-54 (175) | **2.4e-74 (239)** | 4.1e-33 (105) | 2.6e-33 (105) | **2.5e-46 (147)** | 2.7e-28 (89) |
| HMM-Type IIa-3 | 5e-48 (154) | 1.8e-70 (227) | 4.9e-32 (102) | 9.9e-33 (104) | 3.4e-45 (143) | 3.4e-26 (83) |
| HMM-Type IIb | 5.5e-48 (154) | 5.4e-57 (183) | 1.9e-36 (116) | 2.9e-31 (99) | 1.6e-37 (119) | 1.5e-31 (99) |

|  |  |  | **Lamprey Genome** |  |  |
| --- | --- | --- | --- | --- | --- |
| \| Contig ID \| \| --- \| \| Domain Homology‡ \| | 22569.4_6 (586)  TM1 to TM4 | 42790.2_1 (476)  TM1 to TM4 | 31731.1_3 (265)  TM1 to TM4 | 30359.1_1 (909)  TM6 to TM7 | 36401.1_5 (1441)  TM6 to TM7 |
| HMM-Type I | 5.4e-45 (144) | 8.5E-34 (108) | 3.5e-23 (73) | 4.5e-37 (117) | 2.1e-30 (96) |
| HMM-Type IIa-1 | **1.5e-75 (243)** | **6.1E-58 (186)** | **5.4e-41 (131)** | **6.5e-49 (155)** | **1.5e-45 (144)** |
| HMM-Type IIa-2 | 3e-61 (197) | 6.4E-49 (157) | 2e-33 (106) | 4.5e-40 (127) | 3.9e-33 (105) |
| HMM-Type IIa-3 | 1.2e-59 (192) | 3.2E-46 (148) | 8e-32 (101) | 4.8e-39 (123) | 1.6e-33 (106) |
| HMM-Type IIb | 1.1e-54 (175) | 3.5E-45 (144) | 4.1e-31 (99) | 1e-37 (119) | 7.7e-34 (107) |

Numbers represent e-values (with bit scores shown in parentheses) resulting from sequence searches of a HMMER profile database. The sequences used for the searches were identified as the top matches from searches where the HMM profile was a query used to search the genome database (Table 2) from either the little skate (*Leucoraja erinacea*) or lamprey (*Petromyzon marinus*) genome. **Bold values** indicate the best matching HMM model (rows) for each sequence query (columns).

‡ Domain homology indicates the approximate physical position of the HMM, corresponding with Figure 5.
