# Supplementary material for: Dynamic evolution of the GnRH receptor gene family in vertebrates
Source: BMC Evol Biol. 2014 Oct 25;14:215. doi: 10.1186/s12862-014-0215-y (PMC4232701; doi:10.1186/s12862-014-0215-y)
Supplement: Additional file 3: Table S3. — Gar and chimaera gene homology determined with GnRH receptor type-specific HMM profiles using HMMER. [file 12862_2014_215_MOESM3_ESM.docx]

**Table S3.** Gar and chimaera gene homology determined with GnRH receptor type-specific HMM profiles using HMMER.

| **HMM Queries*** | **Gar Genome** | | | **Chimaera Genome** | | |
| --- | --- | --- | --- | --- | --- | --- |
| Sequence ID | LG24_1(2770606) | LG3_2 (15945313) | LG3_1 (15946793) | | AAVX01180425.1 | AAVX01116855.1 |
| Domain Homology‡ | TM1 to TM4 | TM1 to TM4 | TM6 to TM7 | | TM4 to TM5 | TM6 |
| HMM-Type I | 7.6E-39 (119) | 0 (∞) | 0 (∞) | | 2.8e-12 (54.2) | 2.1e-13 (57.5) |
| HMM-Type IIa-1 | 9.4E-58 (180) | 0 (∞) | 0 (∞) | | **1.6e-29 (109.4)** | **2.6e-18 (73.4)** |
| HMM-Type IIa-2 | N/A | 0 (∞) | 0 (∞) | | 3.2e-25 (95.8) | 6.5e-15 (62.8) |
| HMM-Type IIa-3 | 3.2E-56 (176) | 0 (∞) | 0 (∞) | | 6.4e-25 (94.9) | 1.4e-13 (58.4) |
| HMM-Type IIb | **8.2E-75 (235)** | 0 (∞) | 0 (∞) | | 3.2e-20 (79.6) | 2.6e-15 (63.9) |

| **Sequence Queries#** | **Gar Genome** | | | **Chimaera Genome** | | |
| --- | --- | --- | --- | --- | --- | --- |
| Sequence ID | LG24_1(2770606) | LG3_2 (15945313) | LG3_1 (15946793) | | AAVX01180425.1 | AAVX01116855.1 |
| Domain Homology‡ | TM1 to TM4 | TM1 to TM4 | TM6 to TM7 | | TM4 to TM5 | TM6 |
| HMM-Type I | 1.6e-41 (133) | 1.1e-43 (140) | 4.1e-38 (121) | | 2.5E-19 (61) | 1.8E-20 (64) |
| HMM-Type IIa-1 | 1.9e-57 (184) | 8.7e-72 (231) | 1.2e-42 (135) | | **2.9E-36 (115)** | **2.2E-25 (80)** |
| HMM-Type IIa-2 | 2.8e-54 (174) | **2.5e-82 (265)** | **2.9e-54 (173)** | | 5.1E-32 (101) | 6.9E-22 (69) |
| HMM-Type IIa-3 | 6.2e-58 (186) | 3e-71 (229) | 2.2e-45 (144) | | 1.2E-31 (100) | 4.1E-21 (66) |
| HMM-Type IIb | **2.7e-76 (246)** | 5e-58 (186) | 1.8e-42 (134) | | 5.3E-27 (85) | 2.3E-22 (70) |

Numbers represent e-values (with bit scores shown in parentheses) resulting from either: * = an GnRHR type-specific HMM query against either the gar (*Lepisosteus oculatus*) or chimaera (*Callorhinus milii*) genome (as in Table 2), or # = sequence searches of a HMMER profile database (as in Supplemental Table S3). **Bold** values indicate the best HMM model (rows) for each sequence (columns).
