# Supplementary material for: Dynamic evolution of the GnRH receptor gene family in vertebrates
Source: BMC Evol Biol. 2014 Oct 25;14:215. doi: 10.1186/s12862-014-0215-y (PMC4232701; doi:10.1186/s12862-014-0215-y)
Supplement: Additional file 4: Table S4. — Nucleotide sequences of primers used for PCR amplification of GnRH receptor genes in axolotls. [file 12862_2014_215_MOESM4_ESM.docx]

**Table S4:** Nucleotide sequences of primers used for PCR amplification of GnRH receptor genes in axolotls.

| **purpose** | **gene** | **primer sequence (5’ – 3’)** |
| --- | --- | --- |
|  |  |  |
| initial amplification | GnRHR IIa-2, IIa-3, IIb | GTGATGCCNBTGGAKGCBNTKTGGAA |
| initial amplification | GnRHR IIa-2, IIa-3 | CCCARBAGRWARTANGGNGTCCAGCA |
| initial amplification | GnRHR IIa-2, IIa-3 | CTGCHTTWGTYMYNGTRGTVATCAG |
| initial amplification | GnRHR IIa-2, IIa-3 | AKYATCTCHGGSKRRAACCARTACCA |
| initial amplification | GnRHR IIb | AKYATCTCHGGMTGRAACCARTACCA |
| 5’RACE | GnRHR IIa-2 | AGTTCTGAGGAGCCGTGATGGTGACG |
| 5’RACE | GnRHR IIa-2 | GGAACAGGAACAGCTGGGGCAAGGAC |
| 5’RACE | GnRHR IIa-2 | GCCTGGGTGCTATTCATGTCG |
| 5’RACE | GnRHR IIa-3 | AGCTGCCCACCGTAGCACACTGCAC |
| 5’RACE | GnRHR IIa-3 | AGTACACAGGCTGGGAACGGCTCACG |
| 5’RACE | GnRHR IIa-3 | CAAGGGCATGACGATGAACGTAACC |
| 5’RACE | GnRHR IIa-3 | TTTTTCCTGTGCTTGTGGGTGATGG |
| 5’RACE | GnRHR IIb | GTTAGCGCTCTGGATGGTGTGCAGC |
| 5’RACE | GnRHR IIb | AGGATGCTCAGGACCCAAGCCACGC |
| 5’RACE | GnRHR IIb | GCGTAGCACACCACCATGACGCTGAGC |
| 5’RACE | GnRHR IIb | GCCGGCTGCCCCGGTGAAGCTGAAC |
| 3’RACE | GnRHR IIa-2 | CCTGCCGCTGCTCATCATGATCTCC |
| 3’RACE | GnRHR IIa-2 | CCCGGATCCTGCTGGAGATCTCCAG |
| 3’RACE | GnRHR IIa-3 | CCTGCCCCTGCTCATCATGGTGCTC |
| 3’RACE | GnRHR IIa-3 | CCCGCATCCTCCGGGAAATATCCCG |
| 3’RACE | GnRHR IIb | GGCGAGAAGTAAGAGCGACCACATCTCC |
| 3’RACE | GnRHR IIb | GAAGATGACCGTGGTCATCGTGGCG |
| sequencing | GnRHR IIa-2 | CTGATGAGCACTGTGCTGTCCT |
| sequencing | GnRHR IIa-2 | GGAACACCTCCACTGTGTGC |
| sequencing | GnRHR IIa-2 | TGGAGGTAAGCAAGGGTGAC |
| sequencing | GnRHR IIa-2 | ACTTGCTGGTGACGTTTGTG |
| sequencing | GnRHR IIa-2 | AATCCACTTGCAATAAGCGATGC |
| sequencing | GnRHR IIa-2 | GGAGCTCACAATCACAATGCTC |
| sequencing | GnRHR IIa-3 | ATCCTGAGCATTCTCCTAGCTA |
| sequencing | GnRHR IIa-3 | CCGGAAGACGTTTCCTAACCTGG |
| sequencing | GnRHR IIa-3 | CGACTCTTTGGACCATCACC |
| sequencing | GnRHR IIa-3 | CGTCAAGACAATCACTATAGAC |
| sequencing | GnRHR IIa-3 | TGGATTCTCAAACCTCTTGCCTC |
| sequencing | GnRHR IIa-3 | ACCGGACAGGCACACGC |
| sequencing | GnRHR IIb | TCTGATCCTGAGCCTGACG |
| sequencing | GnRHR IIb | ACATCCACCACAGCCTCTTC |
| sequencing | GnRHR IIb | GACGAGCTCTCAGCCAGCTG |
| sequencing | GnRHR IIb | CTGATCAGGACCGCAGTG |
