# Supplementary figures and images for: Dynamic evolution of the GnRH receptor gene family in vertebrates
Source: BMC Evol Biol. 2014 Oct 25;14:215. doi: 10.1186/s12862-014-0215-y (PMC4232701; doi:10.1186/s12862-014-0215-y)

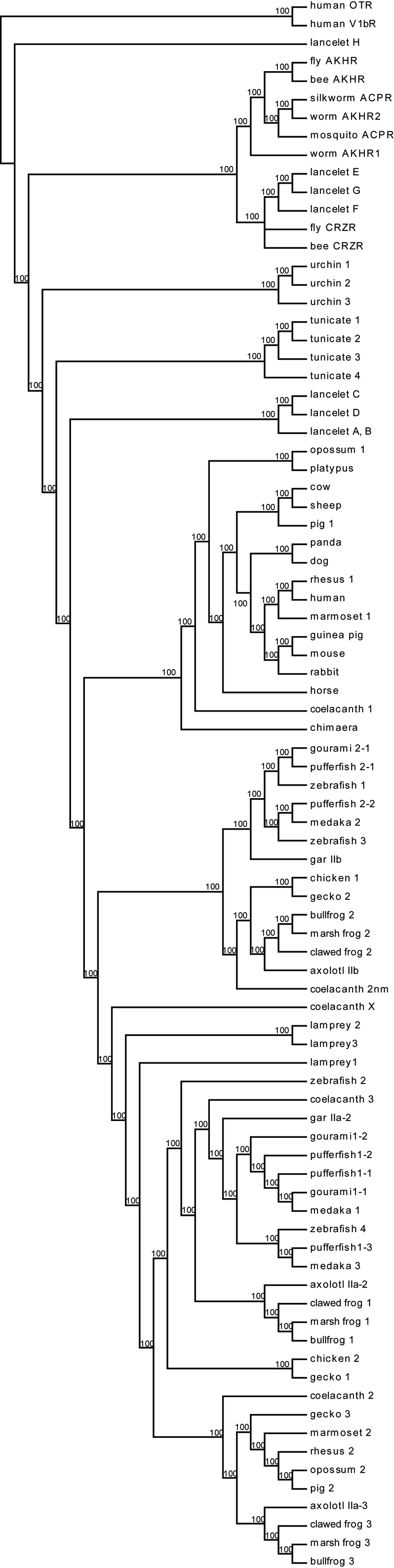

Supplement: Additional file 5: Figure S1. — Maximum likelihood tree illustrating the evolutionary relationships among GnRH receptor homologs from animals, generated using the program RaXML [77] and rooted with the human oxytocin and vasopressin receptors. Numbers above branches indicate bootstrap support values. Two lancelet sequences, A and B, differ by a single amino acid; thus, only one was included in the analysis. ACPR = adipokinetic hormone/corazonin-related peptide receptor; AKHR = adipokinetic hormone receptor; CRZR = corazonin receptor; OTR = oxytocin receptor; V1bR = Type 1b vasopressin receptor. [file 12862_2014_215_MOESM5_ESM.tiff]

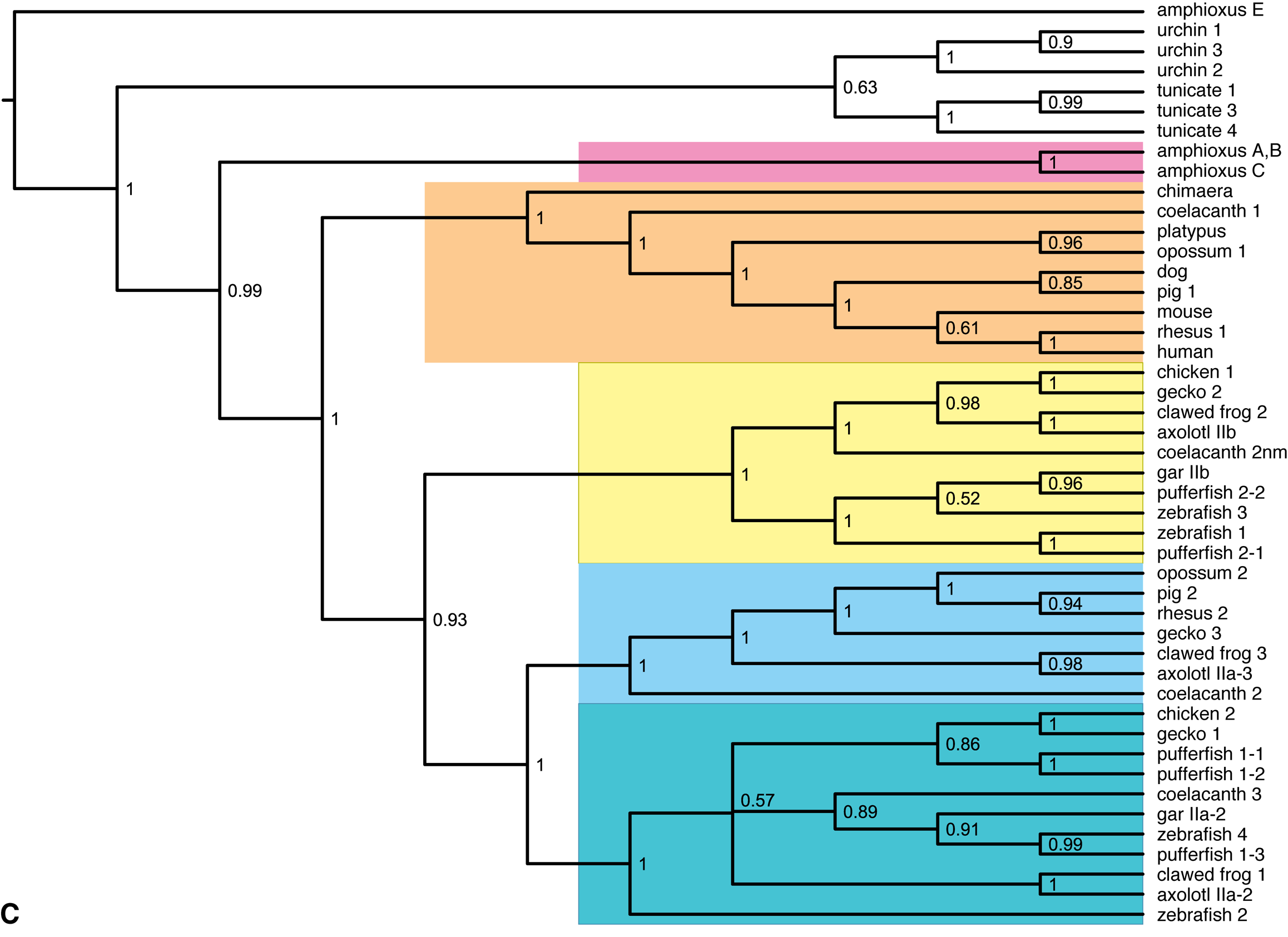

Supplement: Additional file 6: Figure S2. — Cladograms from Bayesian analyses depicting the evolutionary relationships among the genes encoding receptors for GnRH and other peptides; omitting potentially problematic sequences does not substantially alter the topology relative to that shown in Figure 5. Colored backgrounds emphasize strongly-supported, monophyletic subfamilies of GnRH receptors as shown in Figure 5. Numbers indicate the posterior probability support value for the corresponding branch located to the left of the value. Latin names of species and accession numbers for sequences are provided in Additional file 1: Table S1. (a) Tree containing only putative GnRHR sequences from chordates, with all basal taxa removed. (b) As in (a), but also omitting sequences from lampreys. (c) As in (b), but also omitting the coelacanth X sequence. [file 12862_2014_215_MOESM6_ESM.zip › 1940824028133645_add6c.tiff]

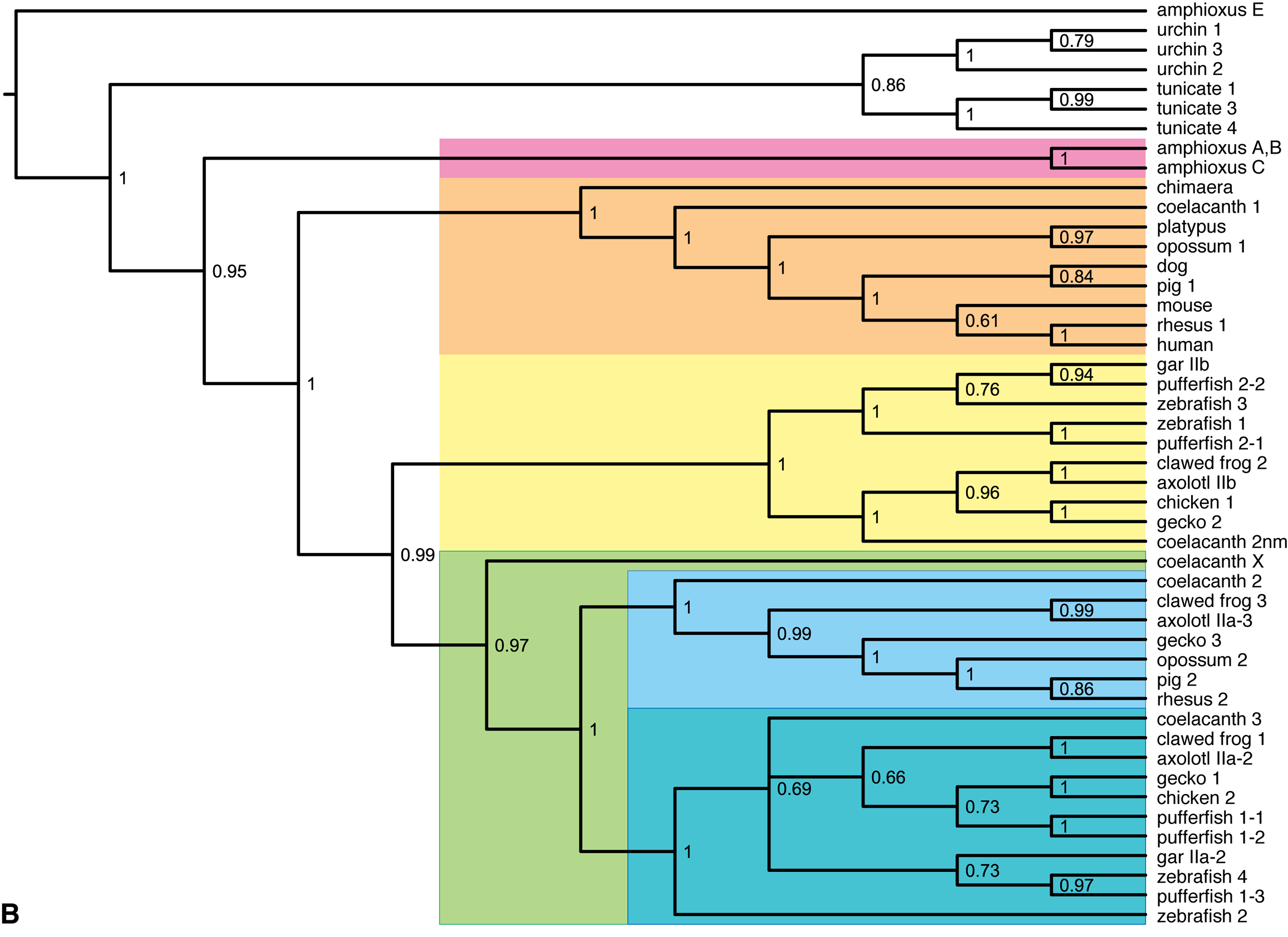

Supplement: Additional file 6: Figure S2. — Cladograms from Bayesian analyses depicting the evolutionary relationships among the genes encoding receptors for GnRH and other peptides; omitting potentially problematic sequences does not substantially alter the topology relative to that shown in Figure 5. Colored backgrounds emphasize strongly-supported, monophyletic subfamilies of GnRH receptors as shown in Figure 5. Numbers indicate the posterior probability support value for the corresponding branch located to the left of the value. Latin names of species and accession numbers for sequences are provided in Additional file 1: Table S1. (a) Tree containing only putative GnRHR sequences from chordates, with all basal taxa removed. (b) As in (a), but also omitting sequences from lampreys. (c) As in (b), but also omitting the coelacanth X sequence. [file 12862_2014_215_MOESM6_ESM.zip › 1940824028133645_add6b.tiff]

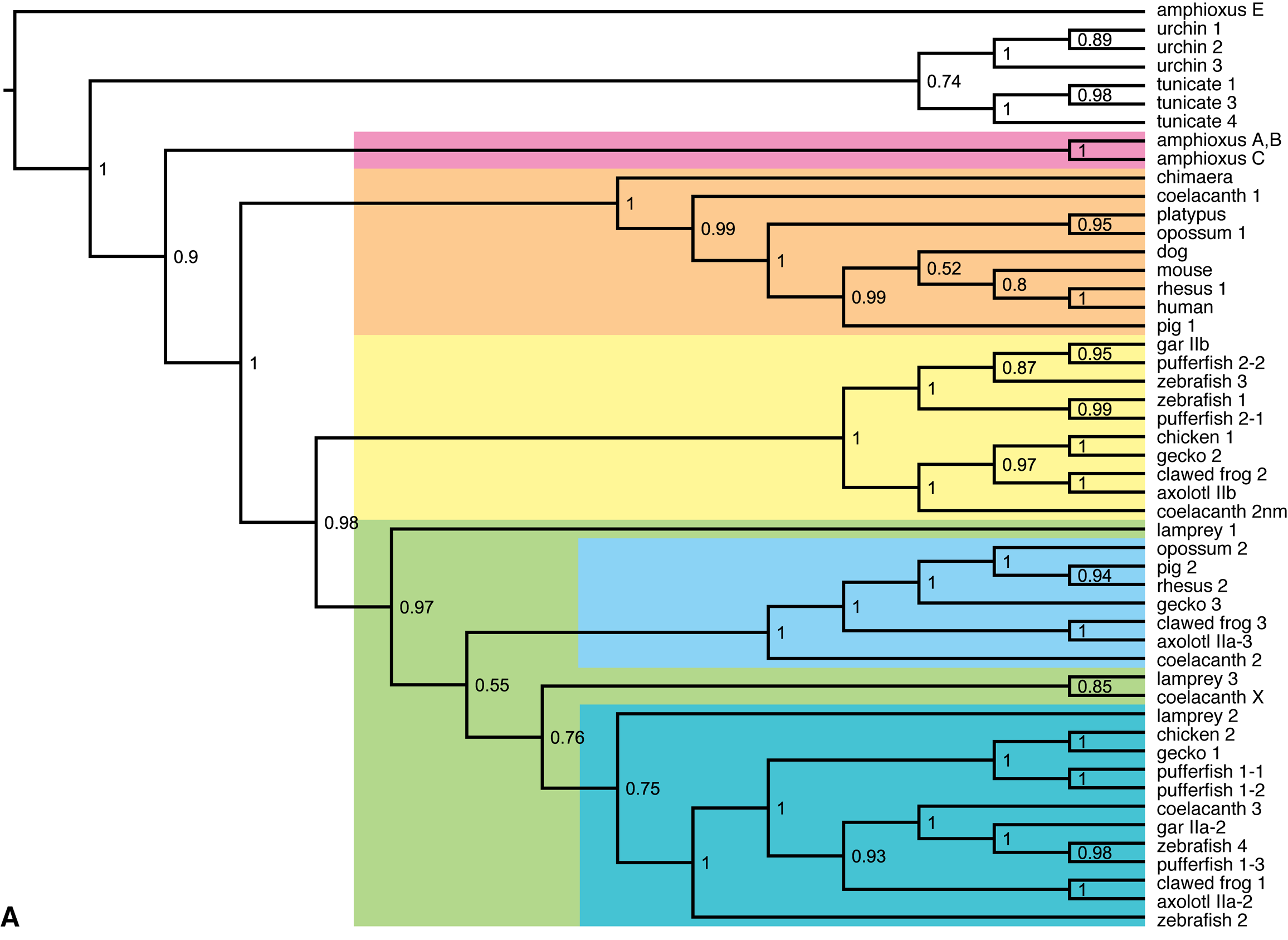

Supplement: Additional file 6: Figure S2. — Cladograms from Bayesian analyses depicting the evolutionary relationships among the genes encoding receptors for GnRH and other peptides; omitting potentially problematic sequences does not substantially alter the topology relative to that shown in Figure 5. Colored backgrounds emphasize strongly-supported, monophyletic subfamilies of GnRH receptors as shown in Figure 5. Numbers indicate the posterior probability support value for the corresponding branch located to the left of the value. Latin names of species and accession numbers for sequences are provided in Additional file 1: Table S1. (a) Tree containing only putative GnRHR sequences from chordates, with all basal taxa removed. (b) As in (a), but also omitting sequences from lampreys. (c) As in (b), but also omitting the coelacanth X sequence. [file 12862_2014_215_MOESM6_ESM.zip › 1940824028133645_add6a.tiff]
